# Effectiveness of eHealth interventions for parents of preterm infants during transition from neonatal intensive care unit-to-home: A systematic review and meta-analysis

**DOI:** 10.1016/j.ijnsa.2026.100684

**Published:** 2026-09-21

**Authors:** Liliana Ferraz, Maria Raul Xavier, Manuel Gameiro, Rafael Vieira, Ana Filipa Cardoso, Daniela Cardoso, Lúcia Paradela, Daniela Dinis, Estela Coutinho, Ana Catarina Maia, Rosa Simões, Ananda Fernandes

**Affiliations:** a Health Sciences Research Unit: Nursing (UICISA:E), Coimbra, Portugal; b School of Nursing, University of Coimbra (ESEUC), Coimbra, Portugal; c Research Centre for Human Development (CEDH), Faculty of Education and Psychology – Universidade Católica Portuguesa, Portugal; d Faculty of Medicine, University of Porto, Porto, Portugal; e Portugal Centre for Evidence Based Practice: a JBI Centre of Excellence, Coimbra, Portugal; f Local Health Unit of Coimbra (ULS Coimbra), Coimbra, Portugal; g XXS - Associação Portuguesa de Apoio ao Bebé Prematuro, Portugal; h European Foundation for the Care of Newborn Infants, Germany

**Keywords:** Ehealth, Early intervention, Neonatal intensive care unit, Preterm infant, Parents, Systematic review, Nursing

## Abstract

**Background:**

Early intervention programmes increasingly use eHealth components to extend family-centred support beyond neonatal intensive care unit discharge. However, their effectiveness on parental and infant health outcomes during the transition from neonatal intensive care unit to home remains unclear.

**Objective:**

To assess the effectiveness of eHealth early intervention programmes supporting parents of preterm infants during the transition from neonatal intensive care unit to home on parental and infant outcomes.

**Design:**

Systematic review and meta-analysis.

**Data sources:**

Medical Literature Analysis and Retrieval System Online (PubMed), Cumulative Index to Nursing and Allied Health Literature (EBSCOhost), Psychological Information Database (ProQuest), Scientific Electronic Library Online, Cochrane Central Register of Controlled Trials (CENTRAL; EBSCOhost), and ClinicalTrials (clinicaltrials.gov) from inception to 20 May 2024, supplemented by grey literature.

**Review methods:**

Eligible randomised controlled trials evaluated eHealth-supported early intervention programmes initiated during neonatal intensive care hospitalisation or shortly after discharge and continuing into the post-discharge period. Random-effects meta-analyses were conducted when at least two trials reported comparable outcomes. Continuous outcomes were pooled using standardised mean differences with 95% confidence intervals.

**Results:**

Seventeen randomised controlled trials involving 2364 parent-infant dyads were included. Pooled analyses favoured eHealth-supported early intervention programmes for depressive symptoms at three months compared with usual care (*standardised mean difference* −0.25; 95% confidence interval −0.43 to −0.08). For parental stress, point estimates at three and six months favoured eHealth-supported programmes, although confidence intervals crossed the null value. Point estimates for anxiety and parenting competence or self-efficacy also generally favoured eHealth-supported programmes, but estimates were imprecise and uncertainty remained. Breastfeeding-related outcomes were mixed and could not be pooled. Infant developmental and health service utilisation outcomes were reported in few trials, were heterogeneous, and precluded quantitative synthesis.

**Conclusion:**

eHealth early intervention programmes may provide small incremental benefits for parental outcomes during the neonatal intensive care unit-to-home transition, particularly in reducing depressive symptoms at three months. Effects on stress, anxiety, and parenting competence or self-efficacy remain uncertain, and evidence for infant outcomes is limited. These findings support the potential role of eHealth as a structured extension of nurse-led and interdisciplinary transitional care, but further robust evaluation is required to clarify effectiveness, equity, implementation, and sustainability.

**Registration:**

PROSPERO CRD42023444721, registered 23 July 2023 and published previously *(protocol publication blinded for peer review).*

**Social media abstract:**

eHealth-supported early intervention may benefit parental wellbeing after Neonatal Intensive Care Unit discharge, particularly for depressive symptoms.

**What is already known**

• Parents of preterm infants have substantial support needs during the transition from neonatal intensive care unit to home, including confidence in caregiving, feeding support, and stress management.
• Early intervention programmes increasingly use eHealth components to extend post-discharge support.
• Evidence on the effectiveness of these programmes on parental and infant outcomes remains limited and heterogeneous.

**What this paper adds**

• eHealth-supported early intervention programmes may provide small incremental benefits for parental depressive symptoms during the transition from neonatal intensive care unit to home.
• eHealth-supported follow-up shows a consistent direction of effect towards lower parental stress beyond active usual care, supporting its potential role in continuity of nursing care after discharge.
• Evidence for infant outcomes remains limited and heterogeneous, highlighting the need for stronger evaluation of infant developmental and health service utilisation outcomes.

## 1 Introduction

Prematurity, defined as birth before 37 completed weeks of gestation, remains a major global public health concern, with incidence estimates ranging between 4% and 16% worldwide. It is a leading cause of mortality among children under five years of age and an important indicator of perinatal health (World Health Organization, 2023a). Advances in neonatal care have improved survival. However, preterm birth remains associated with increased risks of neurodevelopmental impairment, including motor, cognitive, neurosensory, and behavioural sequelae across the life course (Allotey et al., 2018; Robinson et al., 2020). In addition to biological vulnerability, adverse socioenvironmental conditions — such as socioeconomic disadvantage, lower parental education, family dysfunction, and limited access to supportive services — may further influence developmental trajectories and contribute substantially to long-term burdens for individuals, families, and health systems (Bills et al., 2021; Kim et al., 2022).

Parental psychological vulnerability often emerges early during neonatal intensive care unit (NICU) hospitalisation, where prolonged separation, prognostic uncertainty, and exposure to intensive care environments contribute to heightened stress and emotional distress (Givrad et al., 2021; Shillington and McNeil, 2021). These challenges frequently persist during the transition home, when parents assume primary caregiving responsibilities outside the structured support of the NICU (Boykova et al., 2020; Givrad et al., 2021). Preparation for discharge should therefore begin during NICU hospitalisation, as parents progressively assume caregiving roles under professional guidance, and continue after discharge through structured professional follow-up and early supportive interventions (Meleis et al., 2000; Kuo et al., 2012).

### 1.1 Early intervention programmes and developmental foundations

Early intervention programmes, grounded in family-centred care, comprise coordinated services and resources designed to support young children at developmental risk and their families (Carvalho et al., 2019). In neonatal care, relationship-based developmental approaches emphasise active parental involvement and integrative support for premature infants (Als and Gilkerson, 1997). These programmes aim to strengthen parental competence, facilitate caregiving at home, promote infant development, and connect families with community and health services (Carvalho et al., 2019).

The bioecological and systemic model of human development provides a useful conceptual lens, emphasising that outcomes emerge from dynamic interactions among biological, relational, and environmental processes (American Academy of Pediatrics, 2017). This perspective is consistent with developmental neurobiology, which highlights the importance of early experiences in shaping neural plasticity and adaptive functioning (Coughlin, 2021). Within this framework, early intervention programmes commonly include psychosocial support, parental education, and therapeutic input for infant development, with potential effects on parental outcomes such as stress, anxiety, competence and satisfaction, and on infant outcomes including health service utilisation and cognitive, motor, and social development (Benzies et al., 2013).

Evidence from reviews suggests that programmes initiated during the neonatal period and extended after discharge may improve parental and infant outcomes, while also being influenced by contextual and health system factors (Puthussery et al., 2018). Accordingly, transitional neonatal care represents a priority area for integrated follow-up models that maintain continuity and support parental empowerment beyond hospital discharge (Barfield, 2018; Puthussery et al., 2018).

### 1.2 Integration of eHealth into early intervention programmes

Building on this evidence base, the integration of eHealth into early intervention programmes represents an important evolution in neonatal and family care. The COVID-19 pandemic accelerated the adoption of technology-assisted models of care, expanding the use of electronic health approaches to support families during and after NICU hospitalisation. These approaches aim to improve access, continuity, family engagement and professional responsiveness, particularly in contexts affected by geographic or socioeconomic disparities (Lindkvist et al., 2021; Seppänen et al., 2022; Strand et al., 2022).

According to the World Health Organisation (WHO), eHealth refers to the use of information and communication technologies to support health care delivery, communication and service management (World Health Organization, 2023b). Within neonatal transitional care, eHealth may be operationalised through different delivery formats, including mobile health applications, telehealth consultations, messaging systems, web-based platforms and hybrid models combining digital and face-to-face contacts (Sarik et al., 2022; Strand et al., 2022; Haleem et al., 2021). These formats differ not only in technological modality, but also in intensity, interactivity, relational support, behavioural components and professional responsiveness. Consequently, eHealth interventions should not be interpreted as a homogeneous intervention category, as these characteristics may influence engagement, implementation and effectiveness (Dol et al., 2017; Praharaj et al., 2024).

Within early intervention programmes, eHealth approaches can be used to deliver or reinforce parental education, psychosocial support, developmental guidance and care coordination through digital tools (Siani et al., 2017; Dol et al., 2017; Praharaj et al., 2024). Rather than replacing face-to-face care, these approaches may extend the reach of healthcare professionals, enhance continuity after discharge, strengthen parental self-efficacy and support family engagement during the transition from NICU to home (Dol et al., 2017; Praharaj et al., 2024).

### 1.3 Current evidence and research gaps

Recent evidence has highlighted the potential benefits of eHealth approaches in neonatal care contexts. A scoping review by Siani et al. (2017) reported improvements in caregiving knowledge, confidence and perceived support among families of high-risk newborns using eHealth resources during and after NICU hospitalisation. Similarly, communication-focused interventions combining in-person and remote delivery have been associated with reduced parental stress and greater caregiver satisfaction following NICU discharge (Griffith et al., 2022).

Collectively, these findings suggest that eHealth-supported programmes may enhance parental self-efficacy, emotional well-being and continuity of care during the transition home. However, much of the existing literature remains focused on feasibility, acceptability and implementation, with comparatively limited evidence regarding effectiveness on clearly defined parental and infant outcomes.

A preliminary search of PROSPERO, MEDLINE (PubMed), the Cochrane Database of Systematic Reviews and JBI Evidence Synthesis, conducted prior to developing this review, identified only one systematic review addressing eHealth interventions for families of preterm or high-risk infants. That review focused on interventions implemented within NICUs and excluded programmes that continued after discharge (Dol et al., 2017). A more recent systematic review and meta-analysis examined digital health interventions for mothers and high-risk neonates after NICU discharge, but did not include fathers, did not explicitly adopt early intervention programme frameworks, and did not consider interventions initiated during hospitalisation (Praharaj et al., 2024).

Alongside these reviews, randomised controlled trials have evaluated eHealth-supported interventions initiated before discharge and/or continued after discharge, targeting core early intervention components such as psychosocial support, parental education and developmentally oriented guidance for infant care. For example, Phagdol et al. (2023) evaluated a mobile-based educational and support programme initiated before NICU discharge and reported improvements in maternal knowledge and caregiving competence. Taken together, previous scope limitations and emerging trial evidence spanning the NICU-to-home transition support the need for an updated effectiveness-focused synthesis explicitly framed within early intervention models.

Accordingly, this review aimed to synthesise evidence from randomised controlled trials evaluating the effectiveness of eHealth-supported early intervention programmes in improving parental outcomes (stress, anxiety, depressive symptoms, parenting competence or self-efficacy, and breastfeeding self-efficacy, confidence or satisfaction) and infant outcomes (health service utilisation and cognitive, motor, or social development) among families of preterm infants transitioning from the NICU to home.

## 2 Methods

### 2.1 Design and registration

This systematic review and meta-analysis evaluated the effectiveness of eHealth early intervention programmes supporting families of preterm infants during the transition from neonatal intensive care to home. The review was prospectively registered in the International Prospective Register of Systematic Reviews (CRD42023444721), registered on 23 July 2023. The protocol was published prior to study selection (Author et al., 2024).

### 2.2 Eligibility criteria

Studies were selected according to predefined inclusion and exclusion criteria based on the Population, Intervention, Comparator, Outcomes, and Study design framework (Table 1). Eligibility criteria were defined to capture studies evaluating eHealth early intervention programmes supporting families of preterm infants during the transition from neonatal intensive care to home. Randomised controlled trials were eligible if they included preterm infants and their parents and if interventions were initiated during neonatal intensive care hospitalisation or within the first month after discharge. Interventions were required to include at least one eHealth component delivered by health professionals and to incorporate core early intervention elements such as parental education, psychosocial support or developmental guidance. Studies were excluded if interventions were delivered exclusively during hospitalisation without continuity after discharge; if the eHealth component consisted solely of a single contact (for example, a single telephone call, text message or email); or if digital tools were limited to administrative systems, unstructured information provision, or physiological monitoring without a parent-focused educational or supportive component. Eligible studies were required to report parental and/or infant outcomes assessed at baseline and at least one post-intervention or follow-up time point.

**Table 1:** Eligibility criteria using the PICOS format.

PICOS domain	Inclusion criteria
Population	Preterm infants (<37 weeks’ gestation) and their parents (mothers and/or fathers), in hospital, primary care or community settings addressing the transition from NICU to home.
Intervention	eHealth EIPs delivered by health professionals; initiated during NICU or within 1 month after discharge; including ≥1 eHealth component (e.g., web-based platforms, mobile applications, messaging, teleconsultation, video calls); incorporating parental education, psychosocial support, and/or developmental guidance.
Comparator	Standard care (e.g., routine discharge education, scheduled follow-up appointments, or home visits), another eHealth programme, a non-digital intervention, or no intervention.
Outcomes	Parental outcomes (stress, anxiety, depressive symptoms, parenting competence or self-efficacy, breastfeeding-related outcomes, satisfaction or acceptability). Infant outcomes (health service utilisation; cognitive, motor or social development).
Study design	Randomised controlled trials

### 2.3 Deviations from protocol

Two minor deviations from the published protocol were implemented. First, although the protocol initially planned to include quantitative studies of any design, eligibility was ultimately restricted to randomised controlled trials to strengthen internal validity, improve the comparability of effect estimates, and reduce confounding associated with non-randomised designs. The identification of a sufficient number of eligible randomised controlled trials made this methodological refinement feasible without compromising the objectives of the review.

Second, breastfeeding self-efficacy and depressive symptoms were explicitly included as parental outcomes during the screening and eligibility assessment process. Both outcomes were recurrently reported among studies meeting the eligibility criteria and were considered clinically relevant, nursing-sensitive indicators that were conceptually consistent with the review’s predefined scope of parental caregiving and psychological well-being during the transition from the NICU to home. They were subsequently assessed using the same methodological procedures as the other parental outcomes. Because their explicit specification occurred after publication of the protocol, these outcomes were not individually prespecified therein.

These deviations were considered to enhance the completeness and applicability of the review without altering its scope or methodological integrity.

### 2.4 Search methods

A comprehensive search strategy was developed and implemented in collaboration with an experienced health sciences librarian. Searches were conducted from database inception to 20 May 2024 in Medical Literature Analysis and Retrieval System Online (PubMed), Cumulative Index to Nursing and Allied Health Literature (EBSCOhost), Psychological Information Database (ProQuest), Scientific Electronic Library Online, Cochrane Central Register of Controlled Trials (CENTRAL; EBSCOhost), and ClinicalTrials (clinicaltrials.gov).

Unpublished studies and grey literature were sought, through DART-Europe, TDX - Tesis Doctorals en Xarxa (Spain), RCAAP - Repositório Científico de Acesso Aberto de Portugal, and MedNar. Reference lists of included studies and relevant reviews were screened to identify additional eligible studies. Full search strategies for all databases are provided in Supplementary file S1. No date limits were imposed. Studies published in English, Spanish, and Portuguese were included.

### 2.5 Study selection

All records identified through database and grey literature searches were imported into Mendeley v1.19.8 (Mendeley Ltd., Elsevier, The Netherlands), and duplicates were removed. The remaining citations were uploaded to Rayyan QCRI (Qatar Computing Research Institute, Data Analytics, Doha, Qatar) and the titles and abstracts were independently screened by two reviewers against the eligibility criteria. Prior to screening, a calibration exercise was conducted to ensure consistency in the application of eligibility criteria. Full texts of potentially relevant studies were retrieved and assessed independently by the same reviewers. Disagreements at any stage were resolved through discussion or consultation with a third reviewer. When full-text articles were not accessible through institutional subscriptions or interlibrary loans, corresponding authors were contacted directly, resulting in the retrieval of additional studies.

All potentially eligible studies were imported into the JBI System for the Unified Management, Assessment and Review of Information (JBI SUMARI; JBI, Adelaide, Australia) (Munn et al., 2019). Full-text studies that did not meet the inclusion criteria were excluded, and the reasons for exclusion are presented in Supplementary file S2. The study selection process was reported in line with the Preferred Reporting Items for Systematic Reviews and Meta-Analyses (PRISMA 2020) guidelines (Page et al., 2021).

### 2.6 Methodological quality appraisal

The methodological quality of included studies was independently assessed by two reviewers using the Joanna Briggs Institute checklist for randomised controlled trials (Barker et al., 2024). This appraisal comprises 13 items that assess five domains of internal validity: (i) selection and allocation, (ii) intervention administration, (iii) outcome detection and measurement, (iv) participant retention, and (v) statistical conclusion validity. For descriptive purposes, overall methodological quality was pragmatically classified according to the number of checklist items rated “Yes” as higher (≥10/13), moderate (9/13), or lower (≤5/13). Items rated as “Unclear” were not counted as “Yes”. A pilot calibration exercise was conducted on five randomly selected studies before the full assessment to ensure consistency between reviewers. Disagreements were resolved through discussion or consultation with a third reviewer. Methodological quality assessments were used to inform interpretation of findings rather than as criteria for study exclusion.

### 2.7 Data extraction

Data were extracted independently by two reviewers using a standardised data extraction tool implemented within JBI SUMARI (JBI, Adelaide, Australia). A structured, pre-piloted extraction template was used, adapted to the specific characteristics of eHealth-supported early intervention programmes for parents of preterm infants. A pilot calibration exercise was conducted on five randomly selected studies to ensure consistency between reviewers prior to full data extraction. Discrepancies were resolved through discussion and, when necessary, by consultation with a third reviewer. For each included study, the extraction captured key descriptive and methodological characteristics, including: author, year and country; study design and setting; population characteristics and gestational age; intervention type and core components; mode of delivery and professional provider; duration and intensity of the intervention; parental and infant outcomes assessed; outcome measurement instruments; follow-up time points; and main findings. Comparator descriptions were extracted and synthesised narratively. When outcome data were missing, unclear, or incompletely reported, corresponding authors were contacted to request clarification or additional information. A detailed summary of extracted study characteristics is presented in the *Results* section and Supplementary file S3.

### 2.8 Data synthesis and analysis

Continuous outcomes were summarised as means (± standard deviations) at baseline and follow-up, and as change-from-baseline values when reported. Dichotomous outcomes were described using absolute and relative frequencies. Quantitative synthesis followed current JBI guidance for systematic reviews of effectiveness (Aromataris and Munn, 2024). Where appropriate, random-effects meta-analyses were conducted to account for expected clinical and methodological heterogeneity across interventions, settings and outcome measures. Outcomes were grouped according to conceptual proximity, the measurement focus of each instrument, and the outcome domains prespecified in the review protocol. Parenting competence, parenting self-efficacy, perceived maternal parenting self-efficacy, and caregiving knowledge were treated as conceptually related indicators of parental preparedness, and perceived capacity to care for the infant, although these constructs were not considered equivalent.

For continuous outcomes assessed using different instruments within the same broader outcome domain (for example, anxiety, depressive symptoms, stress, and parenting competence or self-efficacy), effects were pooled as standardised mean differences (Borenstein et al., 2021; Deeks et al., 2023). Meta-analyses were performed separately for each outcome and follow-up time point, and pooling was undertaken only when at least two trials provided sufficiently comparable data within each stratum (Deeks et al., 2023). Between-study variance was estimated using the restricted maximum likelihood method, and pooled confidence intervals were calculated using the Hartung-Knapp adjustment, which is recommended for more reliable interval estimation when few studies contribute to a random-effects meta-analysis. However, when only two studies contribute to a pooled estimate, Hartung-Knapp intervals may become very wide and should be interpreted cautiously (IntHout et al., 2014; Röver et al., 2015). Statistical heterogeneity was assessed using the Cochran Q χ² test and quantified with the I² statistic (Higgins et al., 2003). Meta-analyses were conducted using R (R Foundation for Statistical Computing, Vienna, Austria). Given the limited number of studies contributing to most outcome-time point strata, and heterogeneity in intervention models and outcome measures, formal subgroup meta-analyses were not undertaken. When quantitative synthesis was not possible, findings were summarised narratively following established guidance for synthesis without meta-analysis (Aromataris and Munn, 2024).

### 2.9 Certainty of evidence

The certainty of evidence was planned to be assessed at the outcome level using the Grading of Recommendations, Assessment, Development and Evaluation (GRADE) approach (Schünemann et al., 2013). Where appropriate, certainty was formally rated and summarised in a Summary of Findings table developed using GRADEpro GDT (McMaster University, Hamilton, ON, Canada). Certainty was judged across five domains: risk of bias, inconsistency, indirectness, imprecision and publication bias. Two reviewers independently assessed certainty for prioritised outcomes and follow-up time points included in the Summary of Findings table, with disagreements resolved through discussion and, where required, consultation with a third reviewer. Where formal grading was not feasible, findings were synthesised narratively in accordance with GRADE principles.

## 3 Results

### 3.1 Study inclusion

A total of 2103 records were identified through database searches and 721 through trial registers. An additional 10 records were identified through citation searching. After removal of 601 duplicate records and one record marked as ineligible by automation tools, 2232 records were screened by title and abstract. Of these, 2139 records were excluded and 93 reports were sought for retrieval; three reports could not be obtained. Consequently, 92 full-text reports were assessed for eligibility (90 from databases/registers and two additional records identified through citation searching that were not retrieved through database searches). Following full-text assessment, 75 reports were excluded (reasons are provided in Supplementary file S2), most commonly due to protocol-only publications; ineligible population, setting, or focus (for example, term infants or programmes not addressing the NICU-to-home transition); interventions not delivered using eHealth modalities (or delivered exclusively face-to-face); absence of relevant parental or infant outcomes; non-eligible language; or ineligible study design. In line with the protocol deviation, non-randomised studies were excluded. Seventeen randomised controlled trials met the eligibility criteria and were included in the review, representing 2364 parent-infant dyads. The study selection process is shown in Fig. 1 (PRISMA 2020 flow diagram).

**Fig. 1:**
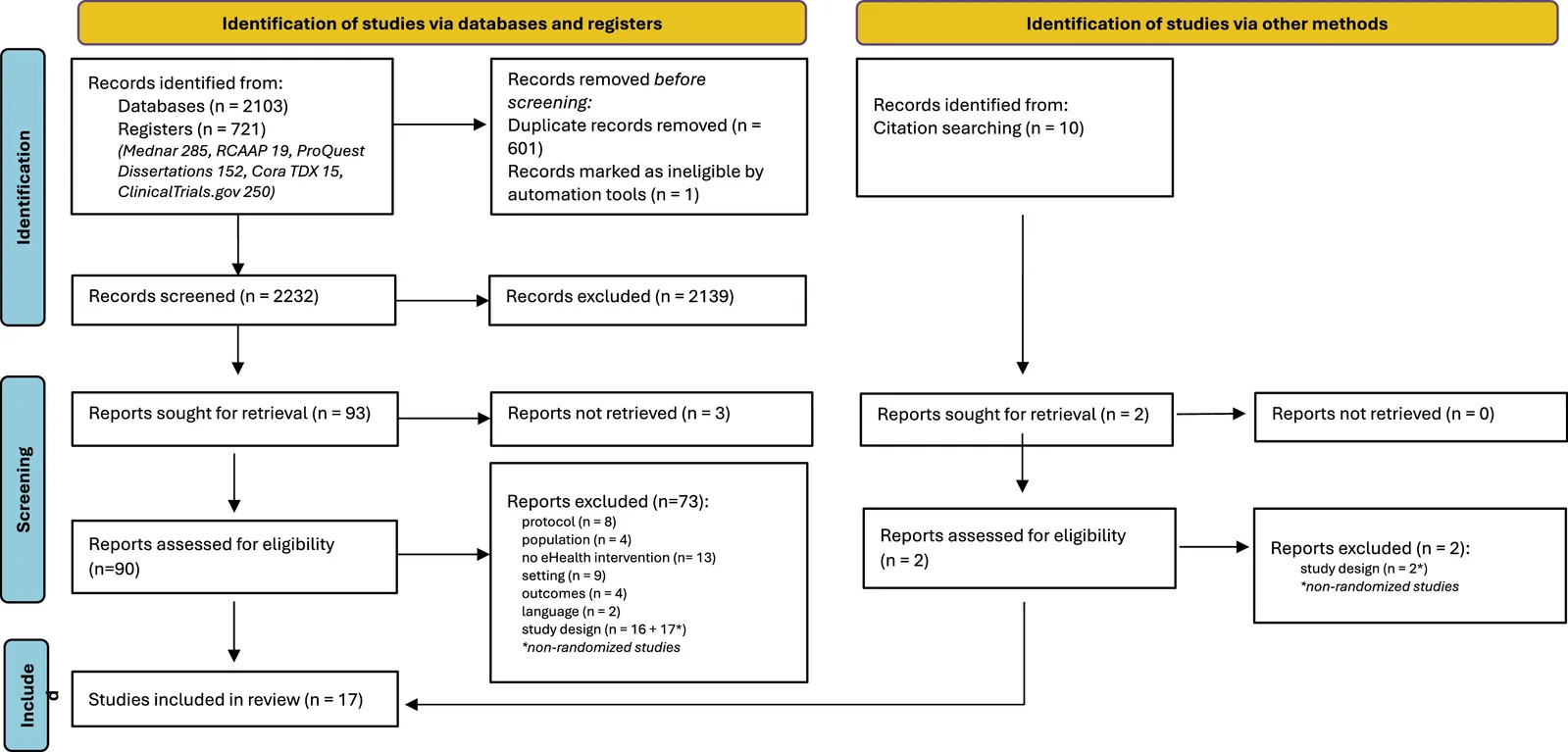
Search results and study selection process (PRISMA 2020 flow diagram).

### 3.2 Methodological quality

Most included trials met the majority of the Joanna Briggs Institute critical appraisal criteria, with 9/17 (52.9%) rated as higher methodological quality (≥10/13 “Yes”), 6/17 (35.3%) as moderate (9/13 “Yes”), and 2/17 (11.8%) as lower quality (≤5/13 “Yes”). Item-level ratings are presented in Table 2.

**Table 2:** Critical appraisal of eligible studies (JBI checklist for randomized controlled trials).

Study	Q1	Q2	Q3	Q4	Q5	Q6	Q7	Q8	Q9	Q10	Q11	Q12	Q13	Result (%)
Asghari et al., 2021	N	N	U	N	N	Y	N	Y	U	Y	Y	Y	U	5/13 = 38.5%
Bahmanpour et al., 2023	N	N	Y	N	N	Y	Y	Y	Y	Y	Y	Y	Y	9/13 = 69.2%
Colditz et al., 2019	Y	Y	Y	N	N	Y	Y	Y	Y	Y	Y	Y	Y	11/13 = 84.6%
Ericson et al., 2018	Y	Y	Y	N	N	Y	U	Y	Y	Y	Y	Y	Y	10/13 = 76.9%
Fratantoni et al., 2022	Y	Y	Y	N	N	Y	N	Y	Y	Y	Y	Y	Y	10/13 = 76.9%
Garfield et al., 2016	Y	Y	Y	N	N	Y	N	Y	Y	N	Y	Y	Y	9/13 = 69.2%
Gund et al., 2013	U	Y	U	N	N	Y	N	U	N	Y	U	U	N	5/13 = 38.5%
Hägi-Pedersen et al., 2022	Y	Y	Y	N	N	Y	N	Y	Y	Y	Y	Y	Y	10/13 = 76.9%
Huang et al., 2024	Y	Y	Y	N	N	Y	Y	Y	Y	Y	Y	Y	Y	11/13 = 84.6%
Jafarzadeh et al., 2019	U	Y	U	N	N	Y	Y	Y	Y	Y	Y	Y	Y	9/13 = 69.2%
Mobbs et al., 2022	Y	Y	Y	N	N	Y	Y	Y	Y	Y	Y	Y	Y	11/13 = 84.6%
Mohammadian et al., 2021	Y	Y	Y	N	N	Y	U	Y	Y	Y	U	Y	Y	9/13 = 69.2%
Neyestani et al., 2017	Y	Y	Y	N	N	Y	N	Y	Y	Y	Y	Y	Y	11/13 = 84.6%
Phagdol et al., 2023	Y	Y	U	N	N	Y	N	Y	Y	Y	Y	Y	Y	9/13 = 69.2%
Robinson et al., 2016	U	U	Y	N	N	Y	Y	Y	Y	Y	Y	Y	Y	9/13 = 69.2%
Schuetz Haemmerli et al., 2024	Y	Y	Y	N	N	Y	Y	Y	Y	Y	Y	Y	Y	11/13 = 84.6%
Yan et al., 2022	Y	Y	Y	N	N	Y	Y	Y	Y	Y	Y	Y	Y	11/13 = 84.6%

Y= yes; N= no; U= unclear. “Result (%)” represents the number of checklist items rated “Yes” (n/13) and the corresponding percentage; unclear ratings were not counted as “Yes”. Overall methodological quality was classified pragmatically as higher (≥10/13 “Yes”), moderate (9/13 “Yes”), or lower (≤5/13 “Yes”).

Q1= Was true randomization used for assignment of participants to treatment groups? Q2= Was allocation to treatment groups concealed? Q3= Were treatment groups similar at baseline? Q4= Were participants blind to treatment assignment? Q5= Were those delivering treatment blind to treatment assignment? Q6= Were outcome assessors blind to treatment assignment? Q7= Were treatment groups treated identically other than the intervention of interest? Q8= Was follow-up complete, and if not, were strategies to address incomplete follow-up utilized? Q9= Were participants analyzed in the groups to which they were randomized? Q10= Were outcomes measured in the same way for treatment groups? Q11= Were outcomes measured in a reliable way? Q12= Was appropriate statistical analysis used? Q13= Was the trial design appropriate, and any deviations from the standard RCT design (individual randomization, parallel groups) accounted for in the conduct and analysis of the trial?.

Across studies, blinding-related items were most frequently rated “No” or “Unclear”, reflecting the behavioural and educational nature of the interventions. Baseline similarity between groups and consistency of outcome measurement were commonly met. Completeness of follow-up and reporting/handling of missing data varied across trials. “Unclear” ratings were retained as reported and were not counted as “Yes” in the descriptive totals. All studies were retained for synthesis, and methodological quality informed interpretation of findings and, where applicable, GRADE certainty ratings.

### 3.3 Characteristics of included studies

#### 3.3.1 Study and participant characteristics

Seventeen randomised controlled trials were included, enrolling 2364 parent-infant dyads across eight countries. Most studies were conducted in Iran (n = 5) and Sweden (n = 3), with additional trials from the United States (n = 2), Australia (n = 2), China (n = 2), Denmark (n = 1), Switzerland (n = 1) and India (n = 1). Sample sizes ranged from 16 to 493 dyads. Most trials focused on mothers of preterm infants (typically 28–36 weeks’ gestation), although several included fathers or both parents. Recruitment commonly occurred during late NICU hospitalisation with follow-up after discharge, or immediately post-discharge, targeting the transition to home. A detailed description of study characteristics, interventions, outcomes and key findings is provided in Supplementary file S3.

#### 3.3.2 Interventions and delivery modes

Across the included randomised controlled trials, eHealth early intervention programmes were delivered through four main modalities. *Telehealth interventions*, including telephone or video support, comprised structured telenursing, scheduled telephone calls, or video consultations providing parental education, monitoring, counselling, and emotional support (Asghari et al., 2021; Ericson et al., 2018; Jafarzadeh et al., 2019; Mohammadian et al., 2021; Robinson et al., 2016; Yan et al., 2022). *Mobile health (mHealth) applications* consisted of smartphone-based interventions offering educational content, symptom or developmental tracking, secure messaging, reminders, and interactive feedback (Bahmanpour et al., 2023; Garfield et al., 2016; Huang et al., 2024; Phagdol et al., 2023). *Web-based platforms* included internet-delivered programmes incorporating structured educational modules, secure communication, parental guidance resources, and telefollow-up components (Neyestani et al., 2017; Gund et al., 2013). *Hybrid models* combined digital follow-up with limited face-to-face components, such as NICU-based education sessions, outpatient consultations, or home visits, alongside remote support (Fratantoni et al., 2022; Schuetz Haemmerli et al., 2024; Hägi-Pedersen et al., 2022; Mobbs et al., 2022; Colditz et al., 2019).

Across studies, eHealth modalities were implemented either as stand-alone interventions or as integrated components of broader post-discharge follow-up programmes, with delivery typically initiated at or shortly after NICU discharge. For analytical purposes, studies were categorised according to the predominant mode of intervention delivery. When multiple digital and non-digital components were present, classification was based on the primary modality through which the intervention was delivered. Nevertheless, these categories should not be interpreted as mutually exclusive or modality-equivalent, because interventions varied substantially in intensity, interactivity, relational support, behavioural components, and professional responsiveness.

#### 3.3.3 Intervention content and dose

The included eHealth interventions combined interrelated components addressing both parental and infant needs (Benzies et al., 2013; Puthussery et al., 2018).

**Psychosocial support** was included in most interventions and was delivered through individualised coaching, scheduled follow-up contacts, or remote interaction with healthcare professionals. These components targeted parental stress, anxiety, coping, and confidence. However, at least one trial (Mobbs et al., 2022) did not include an explicit psychosocial support component, focusing instead on educational and developmental content.

**Parental education** was a consistent component across all interventions and provided structured knowledge and practical guidance related to preterm infant care. Educational content commonly addressed recognition of warning signs, feeding and breastfeeding management, infant care routines, developmental milestones, and strategies to support the transition from hospital to home. Education was delivered through telephone or video consultations, mobile applications, web-based modules, or digital written and audiovisual materials.

**Developmental guidance or therapeutic input** was incorporated in a subset of interventions to support infant development, focusing on promoting responsive parent-infant interaction, early motor and sensory stimulation, and developmentally appropriate caregiving practices. This component was explicitly reported in eight trials (Colditz et al., 2019; Garfield et al., 2016; Gund et al., 2013; Huang et al., 2024; Mobbs et al., 2022; Phagdol et al., 2023; Robinson et al., 2016; Yan et al., 2022), and was commonly supported by video-based modelling, guided parent-infant activities, and individualised feedback delivered remotely via mobile applications, web-based platforms, or telehealth consultations.

In addition, care coordination and follow-up navigation were also embedded in several programmes, including scheduled check-ins, symptom screening, reminders, and facilitation of linkage with community or specialist services, aiming to enhance continuity of care after discharge.

Across studies, the combination, intensity, and duration of these components varied substantially. Interventions were initiated either during the NICU stay or immediately post-discharge and ranged from short-term programmes lasting several weeks to extended follow-up over six months. Contact frequency varied from on-demand support to scheduled weekly or monthly contacts, reflecting heterogeneity in intervention dose and intensity.

#### 3.3.4 Comparators

Most trials compared the eHealth early intervention programmes to standard care, which generally included routine discharge education and standard follow-up. A minority of studies used alternative comparators, such as written information materials or non-digital follow-up contacts without interactive eHealth components.

#### 3.3.5 Outcomes and instruments

Outcomes were grouped into parental and infant domains. Parental outcomes included stress, anxiety, depressive symptoms, parenting competence or self-efficacy, breastfeeding-related outcomes, and satisfaction or acceptability. Within the broader category of parental preparedness, caregiving knowledge was considered conceptually related to, but distinct from, parenting competence and self-efficacy. Infant outcomes included health service utilisation (for example, readmissions and emergency department visits) and developmental outcomes (cognitive, motor or social domains), assessed using validated instruments. Instruments varied across trials, and key measures are summarised in Supplementary file S3.

### 3.4 Review findings

Results are presented by outcome domain. For each domain, findings from the narrative synthesis and, where data permitted, results of the meta-analysis are reported. Detailed characteristics of included studies are summarised in Supplementary file S3, and pooled effects for parental outcomes are presented in Table 3 and corresponding forest plots are shown in Supplementary file S4.

**Table 3:** Pooled effects of eHealth early intervention programmes versus standard care on parental outcomes at specified follow-up timepoints.

Outcome (timepoint)	(k)	(N)	(SMD)	95% CI	I² (%)
Anxiety (3 months)	3	309	−0.15	−0.51 to 0.20	0
Anxiety (6 months)	2	286	−0.16	−0.56 to 0.23	0
Depressive symptoms (1 month)	2	239	−0.29	−2.79 to 2.20	53.7
Depressive symptoms (3 months)	4	398	−0.25	−0.43 to −0.08	0
Depressive symptoms (6 months)	2	286	−0.21	−0.93 to 0.51	0
Parenting self-efficacy/competence (1 month)	3	173	0.32	−0.58 to 1.23	46.9
Parenting self-efficacy/competence (3 months)	4	398	0.25	−0.20 to 0.70	42.5
Parenting self-efficacy/competence (6 months)	2	286	0.17	−2.44 to 2.79	64.7
Parental stress (3 months)	2	295	−0.19	−1.14 to 0.77	0
Parental stress (6 months)	2	286	−0.15	−0.35 to 0.04	0

Random-effects meta-analyses were conducted using the Hartung–Knapp adjustment.

Negative SMD values favour eHealth for anxiety, depressive symptoms and parental stress (lower scores indicate improvement). Positive SMD values favour eHealth for parenting self-efficacy/competence (higher scores indicate improvement).

k= number of studies; N= total number of participants included in each pooled analysis; SMD= standardised mean difference; CI= confidence interval; I²= statistical heterogeneity.

#### 3.4.1 Parental outcomes

***Anxiety*** was reported in six randomised controlled trials (Fratantoni et al., 2022; Huang et al., 2024; Mobbs et al., 2022; Neyestani et al., 2017; Schuetz Haemmerli et al., 2024; Yan et al., 2022) and measured using validated self-report instruments, including the State-Trait Anxiety Inventory (STAI), Generalized Anxiety Disorder-7 (GAD-7), Hospital Anxiety and Depression Scale (HADS), and Self-Rating Anxiety Scale (SAS), across multiple follow-up points from early post-discharge to 6 months. Maternal anxiety was reported in all trials. One trial (Schuetz Haemmerli et al., 2024) also reported paternal anxiety and followed the same direction of effect (Supplementary file S3). Pooled analyses were feasible at 3 and 6 months. Point estimates consistently favoured eHealth-supported programmes; however, effects were small and confidence intervals crossed the null value at both time points. No statistical heterogeneity was detected in pooled strata, although this finding should be interpreted cautiously given the small number of contributing studies (Table 3; Supplementary file S4).

***Depressive symptoms*** were assessed in six randomised controlled trials (Fratantoni et al., 2022; Huang et al., 2024; Mobbs et al., 2022; Neyestani et al., 2017; Schuetz Haemmerli et al., 2024; Yan et al., 2022) using validated self-report instruments, including the Edinburgh Postnatal Depression Scale (EPDS), Center for Epidemiologic Studies Depression Scale (CES-D), Hospital Anxiety and Depression Scale–Depression subscale (HADS-D), and Self-Rating Depression Scale (SDS). Outcomes were measured at multiple time points, ranging from the early post-discharge period (1 month) to 6 months after discharge. All trials reported maternal depressive symptoms, and one trial additionally reported paternal depressive symptoms (Schuetz Haemmerli et al., 2024). Across studies, depressive symptom scores generally tended to be lower in participants receiving eHealth-supported early intervention programmes compared with those receiving usual care (Supplementary file S3). Random-effects meta-analyses favoured eHealth at all pooled time points, with the clearest and most precise pooled effect observed at 3 months (SMD-*standardised mean difference* −0.25, 95% CI −0.43 to −0.08; Table 3; Supplementary file S4). Estimates at 1 month and 6 months were less precise due to the small number of contributing studies and resulting wide confidence intervals (Table 3). Although the direction of effects across individual trials was largely consistent with pooled findings, estimates at several time points remained imprecise due to the limited number of contributing studies.

***Parenting competence or self-efficacy*** was assessed in eight randomised controlled trials (Bahmanpour et al., 2023; Fratantoni et al., 2022; Garfield et al., 2016; Hägi-Pedersen et al., 2022; Huang et al., 2024; Mobbs et al., 2022; Phagdol et al., 2023; Schuetz Haemmerli et al., 2024). Seven trials evaluated perceived parental competence or self-efficacy using validated instruments, including the Parenting Sense of Competence Scale (PSOC), Karitane Parenting Confidence Scale (KPCS), Perceived Maternal Parenting Self-Efficacy Scale (PMPS-E), and the Tool to Measure Parenting Self-Efficacy (TOPSE). One trial (Phagdol et al., 2023) assessed parental knowledge, using a structured questionnaire, reporting higher post-intervention knowledge scores in the intervention group than in standard care. Findings from the narrative synthesis generally favoured eHealth-supported programmes.

Pooled effects at 1, 3, and 6 months also favoured eHealth-supported programmes (Table 3; Supplementary file S4). However, confidence intervals were wide and uncertainty remained substantial, particularly at later follow-up points. Differences in intervention content, interactivity, dose, outcome measures, and assessment timing limited comparability across studies (Supplementary file S3).

***Parental stress*** was assessed in six randomised controlled trials (Asghari et al., 2021; Ericson et al., 2018; Fratantoni et al., 2022; Jafarzadeh et al., 2019; Schuetz Haemmerli et al., 2024; Yan et al., 2022) using conceptually related instruments capturing parental distress and burden including the Perceived Stress Scale (PSS), Parental Stressor Scale: Neonatal Intensive Care Unit (PSS:NICU), Parenting Stress Index–Short Form (PSI-SF), and Zarit Burden Interview (ZBI). Stress outcomes were measured at multiple time points, from the early post-discharge period up to 6 months after discharge. All six trials reported maternal stress outcomes. Across studies, stress scores were generally lower in participants receiving eHealth-supported early intervention programmes compared with those receiving standard care (Supplementary files S3 and S4). Schuetz Haemmerli et al. (2024) was the only trial reporting paternal stress outcomes, which followed the same direction of effect as maternal outcomes. Maternal stress data were used for pooled analyses when both maternal and paternal outcomes were reported, in order to ensure consistency across studies and to avoid unit-of-analysis issues. Pooled estimates at 3 and 6 months favoured eHealth-supported programmes, but confidence intervals crossed the null value at both time points. No statistical heterogeneity was detected in these pooled analyses, although this finding should be interpreted cautiously given the small number of contributing studies (Table 3; Supplementary file S4).

***Breastfeeding-related outcomes*** were reported in three randomised controlled trials (Ericson et al., 2018; Hägi-Pedersen et al., 2022; Mohammadian et al., 2021), using the Breastfeeding Self-Efficacy Scale–Short Form (BSES-SF) and the Maternal Breastfeeding Evaluation Scale (MBES). Outcomes included breastfeeding self-efficacy or confidence, exclusive breastfeeding and breastfeeding satisfaction, assessed at different follow-up time points. Quantitative synthesis was not undertaken because of differences in constructs, endpoints, and timing of assessment. Findings varied across trials: some reported higher breastfeeding self-efficacy or confidence in eHealth-supported groups, whereas effects on exclusive breastfeeding and satisfaction were inconsistent (Supplementary file S3).

***Satisfaction and acceptability*** were assessed using study-specific questionnaires, including measures of satisfaction with home health care, perceived usefulness, communication and support, or service experience. Where reported (Gund et al., 2013; Mobbs et al., 2022; Phagdol et al., 2023; Robinson et al., 2016), parents generally rated eHealth-supported follow-up as acceptable and useful, particularly in relation to timely access to guidance and perceived continuity of care. These findings should be interpreted cautiously because satisfaction and acceptability were not assessed consistently across trials and were measured using study-specific instruments (Supplementary file S3).

#### 3.4.2 Infant outcomes

***Infant developmental outcomes***, including cognitive, motor, and social domains, were assessed in three randomised controlled trials (Colditz et al., 2019; Fratantoni et al., 2022; Huang et al., 2024), using standardised tools or developmental indicators, with follow-up ranging from early infancy to 24 months corrected age. Quantitative synthesis was not undertaken because of heterogeneity in intervention components, developmental domains, assessment instruments, and follow-up time points. Findings varied across trials. Some studies incorporating explicit developmental guidance and coached parent–infant interaction reported favourable cognitive or motor outcomes, whereas findings from programmes primarily focused on parental support were less consistent (Supplementary file S3). Given the small number of studies and variability in outcome assessment, conclusions regarding infant developmental outcomes remain preliminary.

***Health service utilisation*** outcomes were reported in two randomised controlled trials (Fratantoni et al., 2022; Robinson et al., 2016), including hospital readmissions, emergency department visits, and unscheduled healthcare contacts during the post-discharge period. Quantitative synthesis was not undertaken because of differences in outcome definitions, data sources, and event rates. Both trials reported fewer unplanned healthcare contacts among infants in eHealth-supported groups. However, conclusions remain tentative because evidence was derived from only two studies using different measurement approaches (Supplementary file S3).

## 4 Discussion

This systematic review synthesised evidence from 17 randomised controlled trials evaluating eHealth-supported early intervention programmes for families of preterm infants across the NICU-to-home transition. Overall, pooled estimates for parental outcomes generally favoured eHealth-supported programmes, although effects were small and often imprecise.

The clearest pooled signal was observed for depressive symptoms at 3 months. For parental stress and anxiety, point estimates also favoured eHealth-supported programmes where quantitative synthesis was possible, but confidence intervals crossed the null value. Parenting competence or self-efficacy showed a less consistent pattern, with greater variability across studies and follow-up points.

Infant outcomes, including development and health service utilisation, were reported in few trials and could not be pooled because of heterogeneity in outcome definitions, instruments, and follow-up windows. Therefore, findings should be interpreted cautiously, with emphasis on direction, consistency, uncertainty, and clinical plausibility rather than precise estimates of effect magnitude.

### 4.1 Positioning within existing evidence and what this review adds

Earlier reviews of digital interventions in neonatal care primarily focused on intervention feasibility, acceptability, or programmes delivered during NICU hospitalisation without continuity after discharge (Dol et al., 2017; Siani et al., 2017). Subsequently, broader scoping reviews mapped the range of post-discharge support interventions available for families of preterm infants, identifying considerable variation in intervention characteristics while highlighting the need for stronger evidence regarding effectiveness across the NICU-to-home transition (Griffith et al., 2022). More recently, systematic reviews have synthesised digital health interventions supporting mothers and high-risk neonates following discharge, reporting promising findings for selected parental and infant outcomes across a wide range of intervention types, populations, and study designs (Praharaj et al., 2024). However, these reviews primarily synthesised digital health interventions as a technological category. In contrast, the present review specifically examined eHealth-supported early intervention programmes initiated before discharge and/or continuing into the post-discharge period, conceptualising digital technologies as delivery mechanisms embedded within structured transitional care rather than as interventions in themselves. By restricting inclusion to randomised controlled trials, this review provides a more rigorous effectiveness-focused synthesis directly relevant to clinical decision-making during the transition from the neonatal intensive care unit to home.

Since completion of this review, the field has continued to evolve towards a broader conceptual understanding of digitally supported neonatal care. Rather than viewing eHealth simply as a collection of technological tools, recent work conceptualises it as a family-centred model of care in which technology facilitates continuity, communication, professional responsiveness, parental engagement, and access to timely support within routine neonatal services (Paleardi et al., 2026). This perspective also emphasises participatory design, implementation processes, digital literacy, diverse caregiving structures, and contextual integration as key determinants of successful implementation.

The findings of the present review are consistent with this evolving conceptual perspective. They do not suggest that digital platforms alone improve outcomes. Rather, potential benefits appear more likely when eHealth supports structured education, responsive professional contact, continuity across care settings, and sustained parental engagement throughout the transition home. At the same time, this review clarifies where evidence appears comparatively more consistent, particularly regarding parental depressive symptoms, and where important uncertainties remain, especially for parental stress, parenting competence or self-efficacy, infant developmental outcomes, and health service utilisation. These findings reinforce the need for future trials to evaluate not only whether digital components are effective, but also which intervention characteristics, implementation strategies, and contextual factors contribute most to successful transitional care.

### 4.2 Interpreting effects through transitional care and family-centred nursing lenses

The NICU-to-home period can be understood as a developmental and situational transition in which parents move from a highly supported hospital environment to assuming primary responsibility for infant care at home. This transition involves changes in parental roles, confidence, vigilance, decision-making, and perceived preparedness, and may be affected by the continuity, coherence, and accessibility of professional support (Meleis et al., 2000). From a nursing perspective, this is a critical point of care because nurses are often central to discharge preparation, parental education, psychosocial assessment, anticipatory guidance, and coordination between hospital, community, and family contexts.

Family-centred care provides a complementary disciplinary lens by positioning parents as active partners in care rather than passive recipients of information. Partnership, empowerment, shared decision-making, and recognition of family strengths are particularly relevant after NICU discharge, when parents need to interpret infant cues, monitor symptoms, make caregiving decisions, and seek help appropriately (Kuo et al., 2012; Gottlieb, 2016). In this context, eHealth-supported interventions may be understood not simply as technological tools, but as mechanisms for extending relational continuity, professional responsiveness, and guided parental participation beyond the hospital setting.

Within this framework, parental stress, emotional well-being, parenting self-efficacy, and caregiving competence can be interpreted as nursing-sensitive outcomes. These outcomes are plausibly responsive to structured discharge preparation, nurse-led coaching, psychosocial screening, tailored education, and coordinated follow-up. However, because most comparators reflected active usual care, including routine discharge education and follow-up, the observed effects should be interpreted as potential incremental benefits of adding eHealth-supported components to existing care pathways, rather than as evidence that digital support can replace face-to-face care. Outcome-specific considerations and plausible mechanisms are discussed below.

### 4.3 Potential mechanisms and outcome-specific considerations

The pattern of findings suggests that eHealth-supported early intervention programmes may operate less through the digital modality itself and more through the combination of structured education, relational continuity, professional responsiveness, and opportunities for parental coaching. These mechanisms are particularly relevant during the NICU-to-home transition, when parents assume increasing responsibility for infant care while still requiring access to specialist guidance, reassurance, and timely escalation pathways.

For *depressive symptoms*, the clearest pooled effect was observed at 3 months. This finding is clinically relevant because depressive symptoms may follow distinct trajectories from pregnancy through the postpartum period and are influenced by individual, relational, and socioeconomic factors (Molina et al., 2024). Persistent perinatal maternal depression has also been associated with less favourable offspring socioemotional outcomes, suggesting that even modest improvements during early parenting may be meaningful (Morales et al., 2023). In the context of preterm birth, these vulnerabilities may be amplified by prolonged hospitalisation, uncertainty, disrupted early caregiving experiences, and intensive caregiving demands after discharge. The timing of the observed effect may reflect that, immediately after discharge, sleep disruption and caregiving demands limit parents’ capacity to engage fully with intervention components, whereas by 8–12 weeks families may be better positioned to use structured follow-up, mobilise coping resources, and benefit from relational support. This interpretation is consistent with transition theory, where role mastery develops over time (Meleis et al., 2000).

For *stress and anxiety*, point estimates generally favoured eHealth-supported programmes, but effects were small and uncertain. These outcomes are clinically salient in neonatal and paediatric nursing because they may influence parents’ capacity to engage in caregiving, interpret infant cues, participate in follow-up, and respond to warning signs after discharge. Higher parental stress has also been associated with poorer caregiving functioning and reduced parental availability, which may affect early parent–infant interaction after preterm birth (Boykova et al., 2020). However, stress and anxiety were measured using different instruments capturing related but non-equivalent constructs, including NICU-specific stress, general parenting stress, caregiver burden, and general anxiety. This measurement heterogeneity may have contributed to imprecision and supports cautious interpretation of pooled estimates.

*Parenting self-efficacy* and *caregiving competence* showed a less consistent pattern. From a behavioural perspective, *self-efficacy* is shaped by mastery experiences, feedback, modelling, and perceived support, and may therefore be particularly sensitive to the intensity, relational depth, interactivity, and responsiveness of professional input rather than to the eHealth modality itself (Albanese et al., 2019). From a nursing and transitional care perspective, improvements in *caregiving competence* should be understood as part of a broader adaptive process in which parents progressively assume responsibility, consolidate caregiving skills, and develop confidence in recognising and responding to infant needs. Interventions incorporating personalised feedback, bidirectional communication, and clear escalation pathways may therefore be more influential than information-only approaches.

It is also important to recognise that parental competence, self-efficacy, and caregiving knowledge, although related, are conceptually distinct constructs. This heterogeneity limits the specificity of the overall interpretation and reinforces the need for future studies to use conceptually aligned and validated instruments. Within these limitations, the findings suggest that eHealth-supported programmes may offer incremental benefit when added to active usual care. However, their effectiveness is likely to depend on the quality of relational engagement, fidelity of delivery, and integration within transitional care pathways rather than on digital access alone (Meleis et al., 2000; Kuo et al., 2012; Proctor et al., 2011).

*Acceptability* and *satisfaction* are relevant implementation outcomes because they may influence engagement, adherence, and the effective “dose” of digital support received. Across included trials, parents generally valued access to timely guidance and perceived continuity of care. However, satisfaction outcomes were often study-specific and may be vulnerable to selection effects where participation required digital access, confidence, or preference for technology. These considerations reinforce the need for equity-oriented design, including multilingual content, low-bandwidth options, support for families with lower digital literacy, and clear alternatives for those without access (Ross et al., 2016; Greenhalgh et al., 2017).

*Breastfeeding* outcomes varied across trials because studies assessed different indicators, including breastfeeding self-efficacy, exclusivity, and maternal satisfaction. In preterm populations, breastfeeding is also influenced by infant immaturity, feeding difficulties, maternal confidence, and the need for skilled support after discharge. eHealth approaches may therefore be most useful when embedded within integrated breastfeeding care pathways that combine proactive remote support with timely in-person assessment when clinically indicated, rather than functioning as stand-alone educational tools (Leong et al., 2024; Saehoong et al., 2024).

*Infant outcomes* remain the weakest area of evidence. Developmental and health service utilisation outcomes were reported in few trials, measured using heterogeneous instruments and follow-up windows, and could not be pooled. Any developmental effects of early parental support may also be indirect and require longer follow-up to become detectable using standardised assessments. Longer follow-up, harmonised developmental measures, and clearer links between parental outcomes, caregiving processes, and infant outcomes are needed to determine whether benefits for parents translate into sustained developmental or service-level effects.

## 5 Strengths and limitations

This review has several strengths. First, its focus on eHealth-supported early intervention programmes spanning the NICU-to-home interface addresses a clinically important gap in neonatal transitional care. Second, the review followed a prospectively registered and published protocol and applied systematic methods aligned with established guidance for evidence synthesis, including duplicate screening, duplicate extraction, and structured critical appraisal. Third, restricting inclusion to randomised controlled trials strengthened internal validity, and stratification by clinically meaningful follow-up windows, where feasible, supported interpretation of outcome trajectories across the transition home (Deeks et al., 2023; Page et al., 2021).

Several limitations should be considered when interpreting the findings. Outcome measurement and timing varied substantially, and only a subset of outcomes could be pooled at specific time points, limiting precision and comparability across trials.

The use of multiple instruments within the same outcome domain introduced conceptual heterogeneity. For example, anxiety and depressive symptom scales differed in their emphasis on cognitive-affective versus somatic symptoms and in whether they were general or perinatal-specific. In perinatal and NICU contexts, somatic items, such as sleep disturbance or fatigue, may overlap with normative postpartum experiences and intensive caregiving demands, potentially contributing to measurement variability and imprecision.

Similar issues apply to parenting competence and self-efficacy, as the instruments captured related but non-equivalent subdimensions. Caregiving knowledge was considered within the broader conceptual framework of parental preparedness but was reported narratively rather than included in the pooled estimates. This conceptual and measurement heterogeneity should be considered when interpreting the overall pattern of findings.

Accordingly, pooled effects, although estimated using random-effects models and standardised mean differences, should be interpreted cautiously, with greater emphasis on direction, consistency, uncertainty, and clinical plausibility than on precise estimates of effect magnitude.

Intervention models also differed in components, intensity, interactivity, relational support, behavioural content, and professional responsiveness. Reporting of engagement, intervention uptake, adherence, and fidelity was inconsistent across trials, limiting understanding of the effective dose received and potential dose-response relationships. Blinding is rarely feasible in behavioural and educational interventions, and many outcomes were self-reported, increasing susceptibility to performance and detection bias (Schünemann et al., 2013).

Evidence for infant outcomes was sparse and heterogeneous, with variability in outcome definitions, instruments, and follow-up windows precluding quantitative pooling.

Finally, although certainty of evidence was appraised in accordance with GRADE principles where appropriate, several comparisons were characterised by small numbers of contributing studies, wide confidence intervals, and variability in intervention models and outcome measurement. Consequently, certainty was frequently rated down across one or more domains. Conclusions should therefore be interpreted primarily in terms of direction and consistency of effects, uncertainty, and clinical plausibility, rather than as definitive estimates of effectiveness.

## 6 Implications for practice, policy and research

*Practice.* The directional pattern suggests that structured eHealth contacts may provide incremental benefits for parental outcomes when added to active usual care during the NICU-to-home transition, particularly for depressive symptoms and stress. Across included trials, nurse-led contacts were commonly used as a delivery mechanism. However, reporting on integration within routine service structures and long-term sustainability was limited. Therefore, the following points should be understood as practice-relevant considerations rather than direct effectiveness conclusions.

Continuity may be strengthened when eHealth contacts are embedded within discharge pathways and linked to existing follow-up structures rather than operating as parallel systems.

Interactivity, including bidirectional communication, tailored feedback, professional responsiveness and clear escalation pathways, may be more influential than platform choice alone.

Service integration should also include clinical governance mechanisms, including triage criteria, documentation standards and referral for in-person assessment when clinically indicated (Greenhalgh et al., 2017; Proctor et al., 2011). Equity should be treated as a core design and delivery consideration, because smartphone access, connectivity, health literacy and digital literacy may influence reach, engagement and benefit (Boykova et al., 2020; Ross et al., 2016).

*Policy.* Health systems should support digitally enabled transitional follow-up through governance, resourcing and workforce planning, including role clarity for telehealth contacts, secure and interoperable platforms, and documentation and escalation procedures. Equity-oriented policy is essential, as many eHealth models implicitly rely on smartphone access, connectivity, language proficiency and digital confidence. Low-bandwidth alternatives, such as telephone follow-up, and accessible in-person pathways should therefore be retained to mitigate the risk of widening disparities in post-discharge support. Implementation frameworks such as the Nonadoption, Abandonment, Scale-up, Spread, and Sustainability (NASSS) and the Reach, Effectiveness, Adoption, Implementation, and Maintenance (RE-AIM) may assist policy-makers and service leaders in anticipating complexity, monitoring reach and planning sustainable scale-up within organisational constraints (Greenhalgh et al., 2017; Glasgow et al., 2019).

*Research.* Future studies should prioritise adequately powered multicentre randomised controlled trials with harmonised outcome definitions and standardised assessment time points. Fathers, partners and diverse caregiver constellations should be actively included to strengthen the family-centred perspective of eHealth-supported transitional care. Greater conceptual clarity and harmonisation of instruments, particularly those minimising somatic symptom overlap in perinatal contexts, would improve comparability across studies and strengthen interpretability of pooled effects. Trials should report intervention components and delivery processes in sufficient detail, including content, frequency, provider role, responsiveness expectations and escalation rules.

Engagement, adoption, adherence, fidelity and actual use of digital components should be measured systematically, alongside longer follow-up for infant outcomes using standardised developmental measures. Hybrid effectiveness-implementation designs could clarify how context, workforce capacity and organisational determinants shape outcomes when programmes are embedded within routine services (Proctor et al., 2011; Greenhalgh et al., 2017).

Prospective economic evaluation and transparent reporting of resource use should also be incorporated into future trials and service evaluations to inform scalability and sustainability, following the Consolidated Health Economic Evaluation Reporting Standards (CHEERS) 2022 guidance where appropriate (Husereau et al., 2022). Given the strategic positioning of nurses as the primary clinical interface in many interventions, workforce capacity, workload implications, and staffing ratios are likely to be critical determinants of sustainable scale-up (Proctor et al., 2011; Greenhalgh et al., 2017).

## 7 Conclusions

This systematic review and meta-analysis suggests that eHealth-supported early intervention programmes delivered during the NICU-to-home transition may provide small but clinically meaningful benefits, particularly for parental outcomes. The clearest quantitative signal was observed for depressive symptoms at 3 months. For parental stress, point estimates favoured eHealth-supported programmes at 3 and 6 months, although confidence intervals crossed the null value. Effects on anxiety and parenting self-efficacy or competence also generally favoured eHealth, but remained uncertain because of imprecision, small numbers of contributing studies, and heterogeneity across intervention models and outcome measures. Evidence for infant developmental and health service utilisation outcomes remains limited and heterogeneous, precluding pooling and warranting cautious interpretation.

From a nursing perspective, these findings support the potential role of eHealth as a structured extension of transitional care, contributing to continuity, parental coaching, anticipatory guidance and timely reassurance. These mechanisms are central to effective NICU-to-home transitions and are responsive to nurse-led and interdisciplinary intervention. Given that most comparators reflected active usual care, observed effects should be understood as incremental gains rather than substitution for standard discharge preparation or face-to-face follow-up. Future research should clarify which families benefit most, which intervention components are most influential, and how eHealth-supported follow-up can be implemented equitably and sustainably within neonatal and community care pathways.

## Declaration of generative AI and AI-assisted technologies in the manuscript preparation process

During the preparation of this manuscript, the authors used an AI-assisted tool (ChatGPT, OpenAI) solely for language editing and stylistic improvements. All scientific content, analyses, interpretations, and conclusions were developed by the authors, who reviewed and approved the final manuscript and take full responsibility for its content.

## Funding

This research did not receive any specific grant from funding agencies in the public, commercial, or not-for-profit sectors.

## CRediT authorship contribution statement

**Liliana Ferraz:** Writing – review & editing, Writing – original draft, Visualization, Validation, Supervision, Resources, Project administration, Methodology, Investigation, Formal analysis, Data curation, Conceptualization. **Maria Raul Xavier:** Writing – review & editing, Validation, Supervision, Methodology, Investigation, Formal analysis, Data curation, Conceptualization. **Manuel Gameiro:** Writing – review & editing, Validation, Supervision, Methodology, Investigation, Formal analysis, Data curation, Conceptualization. **Rafael Vieira:** Writing – review & editing, Validation, Supervision, Software, Resources, Methodology, Formal analysis. **Ana Filipa Cardoso:** Writing – review & editing, Validation, Resources, Methodology, Investigation, Formal analysis, Conceptualization. **Daniela Cardoso:** Writing – review & editing, Validation, Software, Resources, Methodology, Investigation, Data curation. **Lúcia Paradela:** Writing – review & editing, Validation, Resources, Methodology, Investigation. **Daniela Dinis:** Writing – review & editing, Validation, Resources, Methodology, Investigation. **Estela Coutinho:** Writing – review & editing, Validation, Resources, Methodology, Data curation, Conceptualization. **Ana Catarina Maia:** Writing – review & editing, Methodology, Formal analysis. **Rosa Simões:** Writing – review & editing, Methodology, Formal analysis. **Ananda Fernandes:** Writing – review & editing, Visualization, Validation, Supervision, Software, Resources, Project administration, Methodology, Investigation, Formal analysis, Data curation, Conceptualization.

## Declaration of competing interest

The authors declare that they have no known competing financial interests or personal relationships that could have appeared to influence the work reported in this paper.

## Data Availability

All data supporting the findings of this study are derived from previously published studies and are available within the article and its supplementary materials. The authors are available to provide further information upon request.
