# Supplementary material for: Effectiveness of eHealth interventions for parents of preterm infants during transition from neonatal intensive care unit-to-home: A systematic review and meta-analysis
Source: Int J Nurs Stud Adv. 2026 Sep 21;11:100684. doi: 10.1016/j.ijnsa.2026.100684 (PMC13653947; doi:10.1016/j.ijnsa.2026.100684)
Supplement: Supplementary file 1 [file mmc3.pdf]

# Appendix I: Search strategy<level 1 heading>

## MEDLINE (Pubmed), *search conducted in May 20th, 2024*

| Search | Query | Records retrieved |
|---|---|---|
| **#13 (#3 AND #6 AND #9 AND #12)** | ((((parent*[Title/Abstract] OR father*[Title/Abstract] OR mother*[Title/Abstract]) OR (((("Parents"[Mesh:NoExp]) OR "Fathers"[Mesh]) OR "Mothers"[Mesh]) OR "Single Parent"[Mesh])) AND ((premature*[Title/Abstract] OR preterm*[Title/Abstract] OR preemie*[Title/Abstract]) OR ("Infant, Premature"[Mesh]))) AND ((eHealth[Title/Abstract] OR e-health[Title/Abstract] OR "Social media"[Title/Abstract] OR "Medical apps"[Title/Abstract] OR mHealth[Title/Abstract] OR "communication technology"[Title/Abstract] OR "web cam"[Title/Abstract] OR webcam[Title/Abstract] OR "web camera"[Title/Abstract] OR teleneonatology[Title/Abstract] OR facetime[Title/Abstract] OR skype[Title/Abstract] OR zoom[Title/Abstract] OR teams[Title/Abstract] OR webex[Title/Abstract] OR conferenc*[Title/Abstract] OR telemedicine[Title/Abstract] OR telenursing[Title/Abstract] OR m-health[Title/Abstract] OR mobile[Title/Abstract] OR "internet information technology"[Title/Abstract] OR phone[Title/Abstract] OR smartphone[Title/Abstract] OR iPhone[Title/Abstract] OR iPad[Title/Abstract] OR computer*[Title/Abstract] OR "text message"[Title/Abstract] OR "text messages"[Title/Abstract] OR website*[Title/Abstract] OR video*[Title/Abstract] OR mobile[Title/Abstract] OR web-based[Title/Abstract] OR online[Title/Abstract] OR technolog*[Title/Abstract] OR remote[Title/Abstract] OR telehealth[Title/Abstract] OR Virtual[Title/Abstract] OR Webcast*[Title/Abstract] OR podcast*[Title/Abstract] OR game*[Title/Abstract] OR gamification[Title/Abstract]) OR ((((((((((("Telemedicine"[Mesh:NoExp]) OR "Remote Consultation"[Mesh]) OR "Telenursing"[Mesh])) OR "Telecommunications"[Mesh:NoExp]) OR "Telephone"[Mesh:NoExp]) OR "Cell Phone"[Mesh]) OR "Videoconferencing"[Mesh]) OR "Social Media"[Mesh:NoExp]) OR "Internet"[Mesh:NoExp]) OR "Internet-Based Intervention"[Mesh]) OR "Mobile Applications"[Mesh]) OR "Gamification"[Mesh]))) AND ((Discharge*[Title/Abstract] OR follow-up[Title/Abstract] OR transition*[Title/Abstract] OR Post*[Title/Abstract]) OR (((("Transitional Care"[Mesh]) OR "Continuity of Patient Care"[Mesh:NoExp]) OR "Hospital to Home Transition"[Mesh]) OR "Patient Discharge"[Mesh])) | **753** |
| **#12** | (Discharge*[Title/Abstract] OR follow-up[Title/Abstract] OR transition*[Title/Abstract] OR Post*[Title/Abstract]) OR (((("Transitional Care"[Mesh]) OR "Continuity of Patient Care"[Mesh:NoExp]) OR "Hospital to Home Transition"[Mesh]) OR "Patient Discharge"[Mesh]) | 4,535,979 |
| **#11** | ((("Transitional Care"[Mesh]) OR "Continuity of Patient Care"[Mesh:NoExp]) OR "Hospital to Home Transition"[Mesh]) OR "Patient Discharge"[Mesh] | 58,744 |
| **#10** | Discharge*[Title/Abstract] OR follow-up[Title/Abstract] OR transition*[Title/Abstract] OR Post*[Title/Abstract] | 4,516,463 |
| **#9** | (eHealth[Title/Abstract] OR e-health[Title/Abstract] OR "Social media"[Title/Abstract] OR "Medical apps"[Title/Abstract] OR mHealth[Title/Abstract] OR "communication technology"[Title/Abstract] OR "web cam"[Title/Abstract] OR webcam[Title/Abstract] OR "web camera"[Title/Abstract] OR teleneonatology[Title/Abstract] OR facetime[Title/Abstract] OR skype[Title/Abstract] OR zoom[Title/Abstract] OR teams[Title/Abstract] OR webex[Title/Abstract] OR conferenc*[Title/Abstract] OR telemedicine[Title/Abstract] OR telenursing[Title/Abstract] OR m-health[Title/Abstract] OR mobile[Title/Abstract] OR "internet information technology"[Title/Abstract] OR phone[Title/Abstract] OR smartphone[Title/Abstract] OR iPhone[Title/Abstract] OR iPad[Title/Abstract] OR computer*[Title/Abstract] OR "text message"[Title/Abstract] OR "text messages"[Title/Abstract] OR website*[Title/Abstract] OR video*[Title/Abstract] OR mobile[Title/Abstract] OR web-based[Title/Abstract] OR online[Title/Abstract] OR technolog*[Title/Abstract] OR remote[Title/Abstract] OR telehealth[Title/Abstract] OR Virtual[Title/Abstract] OR Webcast*[Title/Abstract] OR podcast*[Title/Abstract] OR game*[Title/Abstract] OR gamification[Title/Abstract]) OR ((((((((((("Telemedicine"[Mesh:NoExp]) OR "Remote Consultation"[Mesh]) OR "Telenursing"[Mesh])) OR "Telecommunications"[Mesh:NoExp]) OR "Telephone"[Mesh:NoExp]) OR "Cell Phone"[Mesh]) OR "Videoconferencing"[Mesh]) OR "Social Media"[Mesh:NoExp]) OR "Internet"[Mesh:NoExp]) OR "Internet-Based Intervention"[Mesh]) OR "Mobile Applications"[Mesh]) OR "Gamification"[Mesh]) | 1,943,396 |
| **#8** | ((((((((((("Telemedicine"[Mesh:NoExp]) OR "Remote Consultation"[Mesh]) OR "Telenursing"[Mesh])) OR "Telecommunications"[Mesh:NoExp]) OR "Telephone"[Mesh:NoExp]) OR "Cell Phone"[Mesh]) OR "Videoconferencing"[Mesh]) OR "Social Media"[Mesh:NoExp]) OR "Internet"[Mesh:NoExp]) OR "Internet-Based Intervention"[Mesh]) OR "Mobile Applications"[Mesh]) OR "Gamification"[Mesh] | 173,493 |
| **#7** | eHealth[Title/Abstract] OR e-health[Title/Abstract] OR "Social media"[Title/Abstract] OR "Medical apps"[Title/Abstract] OR mHealth[Title/Abstract] OR "communication technology"[Title/Abstract] OR "web cam"[Title/Abstract] OR webcam[Title/Abstract] OR "web camera"[Title/Abstract] OR teleneonatology[Title/Abstract] OR facetime[Title/Abstract] OR skype[Title/Abstract] OR zoom[Title/Abstract] OR teams[Title/Abstract] OR webex[Title/Abstract] OR | 1,884,883 |

| | | |
|---|---|---|
| | conferenc*[Title/Abstract] OR telemedicine[Title/Abstract] OR telenursing[Title/Abstract] OR m-health[Title/Abstract] OR mobile[Title/Abstract] OR "internet information technology"[Title/Abstract] OR phone[Title/Abstract] OR smartphone[Title/Abstract] OR iPhone[Title/Abstract] OR iPad[Title/Abstract] OR computer*[Title/Abstract] OR "text message"[Title/Abstract] OR "text messages"[Title/Abstract] OR website*[Title/Abstract] OR video*[Title/Abstract] OR mobile[Title/Abstract] OR web-based[Title/Abstract] OR online[Title/Abstract] OR technolog*[Title/Abstract] OR remote[Title/Abstract] OR telehealth[Title/Abstract] OR Virtual[Title/Abstract] OR Webcast*[Title/Abstract] OR podcast*[Title/Abstract] OR game*[Title/Abstract] OR gamification[Title/Abstract] | |
| **#6** | (premature*[Title/Abstract] OR preterm*[Title/Abstract] OR preemie*[Title/Abstract]) OR ("Infant, Premature"[Mesh]) | 250,030 |
| **#5** | "Infant, Premature"[Mesh] | 64,895 |
| **#4** | premature*[Title/Abstract] OR preterm*[Title/Abstract] OR preemie*[Title/Abstract] | 234,529 |
| **#3** | (parent*[Title/Abstract] OR father*[Title/Abstract] OR mother*[Title/Abstract]) OR (((("Parents"[Mesh:NoExp]) OR "Fathers"[Mesh]) OR "Mothers"[Mesh]) OR "Single Parent"[Mesh]) | 751,576 |
| **#2** | ((("Parents"[Mesh:NoExp]) OR "Fathers"[Mesh]) OR "Mothers"[Mesh]) OR "Single Parent"[Mesh] | 140,424 |
| **#1** | parent*[Title/Abstract] OR father*[Title/Abstract] OR mother*[Title/Abstract] | 727,359 |

## CINAHL (EBSCO), *search conducted in May 20th, 2024*

| Search ID | Search strategy | Results |
|---|---|---|
| **S13** | S3 AND S6 AND S9 AND S12 | **567** |
| **S12** | S10 OR S11 | (1,041,888) |
| **S11** | (MH "Transitional Care") OR (MH "Continuity of Patient Care") OR (MH "Hospital to Home Transition") OR (MH "Patient Discharge") | (43,825) |
| **S10** | TI ( Discharge* OR follow-up OR transition* OR Post* ) OR AB ( Discharge* OR follow-up OR transition* OR Post* ) | (1,023,704) |
| **S9** | S7 OR S8 | (723,079) |
| **S8** | (MH "Telehealth") OR (MH "Telenursing") OR (MH "Telemedicine") OR (MH "Remote Consultation") OR (MH "Teleconferencing") OR (MH "Internet") OR (MH "Internet-Based Intervention") OR (MH "Social Media+") OR (MH "Email") OR (MH "Telecommunications") OR (MH "Videoconferencing+") OR (MH "Telephone") OR (MH "Cellular Phone") OR (MH "Text Messaging") OR (MH "Smartphone") OR (MH "Instant Messaging") OR (MH "Mobile Applications") OR (MH "Gamification") | (157,179) |
| **S7** | TI ( eHealth OR e-health OR "Social media" OR "Medical apps" OR mHealth OR "communication technology" OR "web cam" OR webcam OR "web camera" OR teleneonatology OR facetime OR skype OR zoom OR teams OR webex OR conferenc* OR telemedicine OR telenursing OR m-health OR mobile OR "internet information technology" OR phone OR smartphone OR iPhone OR iPad OR computer* OR "text message" OR "text messages" OR website* OR video* OR mobile OR web-based OR online OR technolog* OR remote OR telehealth OR Vi ... | (647,340) |
| **S6** | S4 OR S5 | (75,930) |
| **S5** | (MH "Infant, Premature") | (27,389) |
| **S4** | TI ( premature* OR preterm* OR preemie* ) OR AB ( premature* OR preterm* OR preemie* ) | (68,504) |
| **S3** | S1 OR S2 | (284,983) |
| **S2** | (MH "Parents") OR (MH "Adolescent Parents+") OR (MH "Adoptive Parents") OR (MH "Biological Parents") OR (MH "Co-Parents") OR (MH "Fathers") OR (MH "Mothers") OR (MH "Mothers, Working") OR (MH "Multiparas") OR (MH "Primiparas") OR (MH "Single Parent") OR (MH "Adolescent Mothers") OR (MH "Adolescent Fathers") | (101,634) |
| **S1** | TI ( parent* OR father* OR mother* ) OR AB ( parent* OR father* OR mother*) | (257,583) |

## **Cochrane Central Register of Controlled Trials (EBSCO),** *search conducted in May 20th, 2024*

| Search Id | Search Strategy | Results |
|---|---|---|
| **S5** | S1 AND S2 AND S3 AND S4 | **485** |
| **S4** | TI ( Discharge* OR follow-up OR transition* OR Post* ) OR AB ( Discharge* OR follow-up OR transition* OR Post* ) | (693,645) |
| **S3** | TI ( eHealth OR e-health OR "Social media" OR "Medical apps" OR mHealth OR "communication technology" OR "web cam" OR webcam OR "web camera" OR teleneonatology OR facetime OR skype OR zoom OR teams OR webex OR conferenc* OR telemedicine OR telenursing OR m-health OR mobile OR "internet information technology" OR phone OR smartphone OR iPhone OR iPad OR computer* OR "text message" OR "text messages" OR website* OR video* OR mobile OR web-based OR online OR technolog* OR remote OR telehealth OR Vi ... | (209,888) |
| **S2** | TI ( premature* OR preterm* OR preemie* ) OR AB ( premature* OR preterm* OR preemie* ) | (32,845) |
| **S1** | TI ( parent* OR father* OR mother* ) OR AB ( parent* OR father* OR mother*) | (75,668) |

## **APA PsycInfo (ProQuest),** *search conducted in May 20th, 2024*

| Select all | Set | Search | Results |
|---|---|---|---|
| **Select item 6** | **S6** | [S2] AND [S3] AND [S4] AND [S5] | **135** |
| **Select item 5** | **S5** | tiab(Discharge* OR follow-up OR transition* OR Post) OR (MAINSUBJECT.EXACT("Hospital Discharge") OR MAINSUBJECT.EXACT("Continuum of Care")) | 430,667 |
| **Select item 4** | **S4** | tiab(eHealth OR e-health OR "Social media" OR "Medical apps" OR mHealth OR "communication technology" OR "web cam" OR webcam OR "web camera" OR teleneonatology OR facetime OR skype OR zoom OR teams OR webex OR conferenc* OR telemedicine OR telenursing OR m-health OR mobile OR "internet information technology" OR phone OR smartphone OR iPhone OR iPad OR computer* OR "text message" OR "text messages" OR website* OR video* OR mobile OR web-based OR online OR technolog* OR remote OR telehealth OR Virtual OR Webcast* OR podcast* OR game* OR gamification) OR (MAINSUBJECT.EXACT.EXPLODE("Teleconferencing") OR MAINSUBJECT.EXACT("Teleconsultation") OR MAINSUBJECT.EXACT.EXPLODE("Telephone Systems") OR MAINSUBJECT.EXACT.EXPLODE("Mobile Health") OR MAINSUBJECT.EXACT.EXPLODE("Teleconsultation") OR MAINSUBJECT.EXACT.EXPLODE("Social Media") OR MAINSUBJECT.EXACT("Telecommunications Media") OR MAINSUBJECT.EXACT("Telemedicine") OR MAINSUBJECT.EXACT("Internet") OR MAINSUBJECT.EXACT("Gamification")) | 601,649 |
| **Select item 3** | **S3** | MAINSUBJECT.EXACT("Premature Birth") OR tiab(premature* OR preterm* OR preemie*) | 24,285 |
| **Select item 2** | **S2** | tiab(parent* OR father* OR mother*) OR (MAINSUBJECT.EXACT("Single Parents") OR MAINSUBJECT.EXACT("Fathers") OR MAINSUBJECT.EXACT("Parents") OR MAINSUBJECT.EXACT("Homosexual Parents") OR MAINSUBJECT.EXACT("Adoptive Parents") OR MAINSUBJECT.EXACT("Mothers")) | 399,150 |

## **Scielo,** *search conducted in May 20th, 2024*

| Search strategy | Results |
|---|---|
| **(ab:(premature* OR preterm* OR preemie*)) AND (ab:(parent* OR father* OR mother*)) AND (ab:(eHealth OR e-health OR "Social media" OR "Medical apps" OR mHealth OR "communication technology" OR "web cam" OR webcam OR "web camera" OR teleneonatology OR facetime OR skype OR zoom OR teams OR webex OR conferenc* OR telemedicine OR telenursing OR m-health OR mobile OR "internet information technology" OR phone OR smartphone OR iPhone OR iPad OR computer* OR "text message" OR "text messages" OR website* OR video* OR mobile OR web-based OR online OR technolog* OR remote OR telehealth OR Virtual OR Webcast* OR podcast* OR game* OR gamification)) AND (ab:(Discharge* OR follow-up OR transition* OR Post*))** | **163** |

## **MedNar,** *search conducted in May 20th, 2024*

| Search | | Results |
|---|---|---|
| | eHealth OR e-health OR "Social media" OR "Medical apps" OR mHealth OR "communication technology" OR "web cam" OR webcam OR "web camera" OR teleneonatology OR facetime OR skype OR zoom OR teams OR webex OR conferenc* OR telemedicine OR telenursing OR m-health OR mobile OR "internet information technology" OR phone OR smartphone OR iPhone OR iPad OR computer* OR "text message" OR "text messages" OR website* OR video* OR mobile OR web-based OR online OR technolog* OR remote OR telehealth OR Virtual OR Webcast* OR podcast* OR game* OR gamification / Title: (premature* OR preterm* OR preemie*) AND (Discharge* OR follow-up OR transition* OR Post*) AND (parent* OR father* OR mother*) | **285** |

## **TDX Tesis Docttorals en Xarxa,** *search conducted in May 20th, 2024*

| Search | Results |
|---|---|
| **premature* AND discharge** | 15 |

## **RCAAP_Repositorios Cientificos,** *search conducted in May 20th, 2024*

| Search | | Results |
|---|---|---|
| **#1 AND #2** | premature* AND discharge | 19 |
| **#2 Tittle:** | premature* AND | |
| **#1 Tittle:** | discharge | |

## **Clinical Trials (EBSCO),** ***search conducted in May 20th, 2024***

| Search | | Results |
|---|---|---|
| | premature* OR preterm* OR preemie* \| Other terms: Discharge* OR follow-up OR transition* OR Post* \| eHealth OR e-health OR "Social media" OR "Medical apps" OR mHealth OR "communication technology" OR "web cam" OR webcam OR "web camera" OR teleneonatology OR facetime OR skype OR zoom OR teams OR webex OR conferenc* OR telemedicine OR telenursing OR m-health OR mobile OR "internet information technology" OR phone OR smartphone OR iPhone OR iPad OR computer* OR "text message" OR "text messages" OR website* OR video* OR mobile OR web-based OR online OR technolog* OR remote OR telehealth OR Virtual OR Webcast* OR podcast* OR game* OR gamification | **250** |
