# Supplementary material for: Effectiveness of eHealth interventions for parents of preterm infants during transition from neonatal intensive care unit-to-home: A systematic review and meta-analysis
Source: Int J Nurs Stud Adv. 2026 Sep 21;11:100684. doi: 10.1016/j.ijnsa.2026.100684 (PMC13653947; doi:10.1016/j.ijnsa.2026.100684)
Supplement: Supplementary file 2 [file mmc4.pdf]

Appendix III: Studies ineligible following full-text review

Exclusion reason	Studies excluded	n
**Protocol only:**<br>*Articles reporting only trial design or registry information, without published results for parental or infant outcomes.*	Baraldi, E., Allodi, M. W., Löwing, K., Smedler, A.-C., Westrup, B., & Ådén, U. (2020). Stockholm preterm interaction-based intervention (SPIBI): Study protocol for an RCT of a 12-month parallel-group post-discharge program for extremely preterm infants and their parents. BMC Pediatrics, 20, 49. https://doi.org/10.1186/s12887-020-1934-4<br><br>Ericson, J., Eriksson, M., Hellström-Westas, L., Hagberg, L., Hoddinott, P., & Flacking, R. (2013). The effectiveness of proactive telephone support provided to breastfeeding mothers of preterm infants: Study protocol for a randomized controlled trial. BMC Pediatrics, 13, 73. https://doi.org/10.1186/1471-2431-13-73<br><br>Ginsberg, K. H., Alsweiler, J., Rogers, J., Cavadino, A., Douglas, M., & Serlachius, A. (2024). A digital Acceptance and Commitment Therapy and education intervention targeting stress of parents and caregivers with preterm babies in the neonatal intensive care unit: A randomised controlled cluster trial protocol. Contemporary Clinical Trials, 140, 107519. https://doi.org/10.1016/j.cct.2024.107519<br><br>Glazebrook, C., Redsell, S., Johnson, S., Beer, C., Budge, H., & Wharrad, H. (2011). Helping our premature infants on to better motor skills (HOP-ON): Development and evaluation of a parenting intervention to promote motor development in infants born very preterm (Protocol version 1.8, December 13, 2011). University of Nottingham<br><br>Hägi-Pedersen, M.-B., Norlyk, A., Dessau, R., Stanchev, H., & Kronborg, H. (2017). Multicentre randomised study of the effect and experience of an early inhome programme (PreHomeCare) for preterm infants using video consultation and smartphone applications compared with inhospital consultations. *BMJ Open, 7*, e013024. https://doi.org/10.1136/bmjopen-2016-013024<br><br>Nayak, B. S., Lewis, L. E., Margaret, B., Bhat, R. Y., & D'Almeida, J. (2019). Randomized controlled trial on effectiveness of mHealth-based preterm home care program on developmental outcomes of preterms: Study protocol. Journal of Advanced Nursing, 75, 452–460. https://doi.org/10.1111/jan.13879<br><br>Saarinen, T., Ylijoki, M., Lehtonen, L., & Rautava, P. (2023). Web-based follow-up tool (ePIPARI) of preterm infants: Study protocol for feasibility and performance. BMC Pediatrics, 23(1), 413. https://doi.org/10.1186/s12887-023-04226-4<br><br>Tooten, A., Hoffenkamp, H. N., Hall, R. A., Winkel, F. W., Eliëns, M., Vingerhoets, A. J. J. M., & van Bakel, H. J. A. (2012). The effectiveness of video interaction guidance in parents of premature infants: A multicenter randomised controlled trial. BMC Pediatrics, 12, 76. https://doi.org/10.1186/1471-2431-12-76	8
**Population not eligible**<br>*Population does not correspond to parents of preterm infants or preterm infants in NICU-to-home transition (e.g., term infants, general pediatric samples, pregnant women, adult patients, staff nurses).*	Binder-Olibrowska, K. W., Godycki-Cwirko, M., & Wrzesinska, M. A. (2022). "To be treated as a person and not as a disease entity": Expectations of people with visual impairments towards primary healthcare. International Journal of Environmental Research and Public Health, 19, 13519. https://doi.org/10.3390/ijerph192013519<br><br>Flanagan, A., Mukherjee, S., Urban, M., Sweeney, K., Prero, B., & Viverito, N. (2022). The effect of robotic-assisted gait training for children with neuromotor disorders in a clinical setting: A case series. Developmental Medicine & Child Neurology, 64(Suppl. 4), 80–138. https://doi.org/10.1111/dmcn.15356<br><br>Hagen, C., Boyer, E., Summers, R., Lykken, L., Barney, C., & Symons, F. (2020). A case-control pilot study assessing nociceptive flexion reflex in females with cerebral palsy. Developmental Medicine & Child Neurology, 62(Suppl. 3), 79. https://doi.org/10.1111/dmcn.14662<br><br>Shinde, S., Madhale, M. D., Panari, H., & Shanmugam, R. (2021). Effectiveness of video-assisted teaching programme on knowledge regarding care of children on mechanical ventilator among staff nurses working in pediatric intensive care units of selected hospitals. Annals of R.S.C.B., 25(4), 7420–7436	4
**No eHealth intervention**<br>*Early intervention or discharge programme delivered without an explicit eHealth/mHealth/telehealth component (only in person, passive videos or printed material), or technology used only for assessment (e.g., video for motor scoring, without interaction with parents).*	Barlow, J., Sembi, S., & Underdown, A. (2016). Pilot RCT of the use of video interactive guidance with preterm babies. Journal of Reproductive and Infant Psychology, 34(5), 511–524. https://doi.org/10.1080/02646838.2016.1217404<br><br>Carty, C. L., Soghier, L. M., Kritikos, K. I., Tuchman, L. K., Jiggetts, M., Glass, P., Streisand, R., & Fratantoni, K. R. (2018). The Giving Parents Support Study: A randomized clinical trial of a parent navigator intervention to improve outcomes after neonatal intensive care unit discharge. Contemporary Clinical Trials, 70, 117–134. https://doi.org/10.1016/j.cct.2018.05.004<br><br>Dusing, S. C., Brown, S. E., Van Drew, C. M., Thacker, L. R., & Hendricks-Muñoz, K. D. (2015). Supporting play exploration and early development intervention from NICU to home: A feasibility study. Pediatric Physical Therapy, 27(3), 267–274. https://doi.org/10.1097/PEP.0000000000000161<br><br>Ericson, J., Lampa, E., & Flacking, R. (2021). Breastfeeding satisfaction post hospital discharge and associated factors – A longitudinal cohort study of mothers of preterm infants. International Breastfeeding Journal, 16, 28. https://doi.org/10.1186/s13006-021-00374-4<br><br>Fan, J., He, R., He, S., Yang, M., Tao, X., Zhou, M., Gao, X., Yu, W., & Wang, J. (2024). Effectiveness of a home-based, post-discharge early intervention program for very preterm infants in reducing parental	13

	stress: A randomized controlled trial. BMC Public Health, 24, 2476. https://doi.org/10.1186/s12889-024-19969-7<br><br>Karbandi, S., Momenizadeh, A., Heidarzadeh, M., Mazlom, S. R., & Hassanzadeh, M. H. (2015). Effects of maternal empowerment program on the length of hospitalization and readmission rate of premature infants. Journal of Babol University of Medical Sciences, 17(10), 28–33. https://doi.org/10.22038/jbums.2015.5448<br><br>Khoshnavay Fomani, F., Mirlashari, J., Sharif Nia, H., Begjani, J., Khoshakhlagh, F., Shiri, M., & Teymuri, E. (2022). Exploring the effects of self-reflection practice on cognitive emotion regulation and resilience among mothers of premature neonates. Nursing Practice Today, 9(3), 211–220 https://doi.org/10.18502/npt.v9i3.10223<br><br>McQueen, D., Lakes, K. D., Rich, J., Vaughan, J., Hayes, G., Cooper, D., & Olshansky, E. (2013). Feasibility of a caregiver-assisted exercise program for preterm infants. Journal of Perinatal and Neonatal Nursing, 27(2), 184–192. https://doi.org/10.1097/JPN.0b013e31828b244a<br><br>Meyer, E. C., Garcia Coll, C. T., Lester, B. M., Boukydis, C. F. Z., McDonough, S. M., & Oh, W. (1994). Family-based intervention improves maternal psychological well-being and feeding interaction of preterm infants. Pediatrics, 93(2), 241–246<br><br>Popescu, O. A. M. (2021). A mixed methods study of the follow-up of extremely preterm babies in the North East of England [Doctor of Medicine thesis, Newcastle University]<br><br>Safran, C., Pompilio-Weitzner, G., Emery, K. D., & Hampers, L. (2005). A Medicaid eHealth program: An analysis of benefits to users and nonusers. AMIA Annual Symposium Proceedings, 2005, 659–663<br><br>Teti, D. M., Black, M. M., Viscardi, R., & Glass, P. (2009). Intervention with African American premature infants: Four-month results of an early intervention program. Journal of Early Intervention, 31(2), 146–166. https://doi.org/10.1177/1053815109331864<br><br>Wheeden, C. A. (1995). The effects of a comprehensive early intervention program on the quality of maternal interactive behavior with low birthweight, premature infants [Doctoral dissertation, University of Miami]	
**Setting/context not eligible**<br>*Interventions carried out exclusively in the NICU context, without structured continuity after discharge; or programmes in other contexts unrelated to the NICU-home transition.*	Collette, K., Feeley, N., Galipeau, R., & Lebel, V. (2023). Acceptability and feasibility of a digital educational intervention designed to improve the psychological well-being of parents with a preterm infant at the neonatal intensive care unit: A pilot project. Early Human Development, 176, 105717. https://doi.org/10.1016/j.earlhumdev.2023.105717<br><br>Franck, L. S., Gay, C. L., Hoffmann, T. J., Kriz, R. M., Bisgaard, R., Cormier, D. M., Joe, P., Lothe, B., & Sun, Y. (2022). Neonatal outcomes from a quasi-experimental clinical trial of Family Integrated Care versus Family-Centered Care for preterm infants in U.S. NICUs. BMC Pediatrics, 22(1), 674. https://doi.org/10.1186/s12887-022-03732-1<br><br>Hayashi, M., Huber, K., Rankin, C., Boyajian, B., Martinez, A., Grover, T., & Roosevelt, G. (2022). BLOSSoM: Improving human milk provision in preterm infants through texting support. Pediatric Quality & Safety, 7, e600. https://doi.org/10.1097/PQ9.0000000000000600<br><br>Khanjari, S., Bell, E. F., Mohagheghi, P., Sadeghi, L. S., Haghani, S., & Rokhsatichenijani, E. (2022). The effect of family-centered education on the quality of life of the parents of premature infants. Journal of Neonatal Nursing, 28(6), 407–412. https://doi.org/10.1016/j.jnn.2021.10.005<br><br>Fomani, F. K., Mirlashari, J., Nia, H. S., Begjani, J., Khoshakhlagh, F., Shiri, M., & Teymuri, E. (2022). Exploring the effects of self-reflection practice on cognitive emotion regulation and resilience among mothers of premature neonates. Nursing Practice Today. https://doi.org/10.18502/npt.v9i3.10223<br><br>Kongsaenkaew, K., Rungamornrat, S., & Payakkaraung, S. (2024). Enhancing maternal self-efficacy in caring for preterm infants with ventilator through a telehealth program: A randomized controlled trial. Pacific Rim International Journal of Nursing Research, 28(1), 88–102. https://doi.org/10.60099/prijnr.2024.264464<br><br>Kwong, A. K. L., Olsen, J., Eeles, A., Einspieler, C., Lee, K., Doyle, L. W., Cheong, J. L. Y., & Spittle, A. J. (2019). Occurrence of and temporal trends in fidgety general movements in infants born extremely preterm/extremely low birthweight and term-born controls. Early Human Development, 135, 11–15. https://doi.org/10.1016/j.earlhumdev.2019.05.018<br><br>Nambiar, S. K., Nayak, S., & Sujatha, R. (2020). Effect of nurse-led programme on knowledge regarding identification of warning signs in low birth weight babies among post-natal mothers in selected hospitals of Mangaluru. Nursing Journal of India, 111(2), 58–63. https://doi.org/10.48029/NJI.2020.CXI202<br><br>Steinhardt, A., Hinner, P., Kühn, T., Roehr, C. C., Rüdiger, M., & Reichert, J. (2015). Influences of a dedicated parental training program on parent–child interaction in preterm infants. Early Human Development, 91(3), 205–210. https://doi.org/10.1016/j.earlhumdev.2015.01.012	9
**Outcomes not relevant**<br>*Does not report parental or infant outcomes aligned with the review question*	Derwig, M., Lindkvist, R.-M., Kristensson Hallström, I., Johnsson, B. A., & Stenström, P. (2023). eHealth usage among parents to premature or surgically treated neonates: Associations with eHealth literacy, healthcare satisfaction or satisfaction with an eHealth device. BMC Pediatrics, 23, 524. https://doi.org/10.1186/s12887-023-04340-3	4

(stress, anxiety, depression, competence, breastfeeding/self-efficacy, satisfaction, service utilisation, development). Includes studies focused solely on costs, consultation rates, or organisational indicators.	Dowling, D. A., Barsman, S. G., Forsythe, P., & Damato, E. G. (2018). Caring about Preemies' Safe Sleep (CaPSS): An educational program to improve adherence to safe sleep recommendations by mothers of preterm infants. Journal of Perinatal & Neonatal Nursing, 32(4), 366–372. https://doi.org/10.1097/JPN.0000000000000345<br><br>Lindkvist, R.-M., Sjöström-Strand, A., Landgren, K., Johnsson, B. A., Stenström, P., & Kristensson Hallström, I. (2021). "In a way we took the hospital home": A descriptive mixed-methods study of parents' usage and experiences of eHealth for self-management after hospital discharge due to pediatric surgery or preterm birth. International Journal of Environmental Research and Public Health, 18(12), 6480. https://doi.org/10.3390/ijerph18126480<br><br>Watson, L., Woods, C. W., Cutler, A., DiPalazzo, J., & Craig, A. K. (2023). Telemedicine improves rate of successful first visit to NICU follow-up clinic. Hospital Pediatrics, 13(1), 3–8. https://doi.org/10.1542/hpeds.2022-006874	
**Study design not eligible**<br>*Non-randomised studies or studies without a comparison group (pre-post, case series, feasibility studies, qualitative/mixed descriptive studies), as well as observational and quasi-experimental studies excluded. It also includes pilot RCTs without an interactive eHealth component.*	Ahn, H. Y., Ko, H. J., & Jo, H. J. (2023). Development and effects of mobile-application-based parenting support program for premature infants' mothers. Healthcare, 11(19), 2639. https://doi.org/10.3390/healthcare11192639<br><br>Albayrak, B., Cordier, L. J., Greve, S., Teschler, U., Dathe, A.-K., Felderhoff-Müser, U., & Hüning, B. M. (2023). Feasibility of video consultation for preterm neurodevelopmental follow-up care during the COVID-19 pandemic: Cohort study. JMIR Pediatrics and Parenting, 6, e40940. https://doi.org/10.2196/40940<br><br>Banerjee, J., Aloysius, A., Mitchell, K., Silva, I., Rallis, D., Godambe, S. V., & Deierl, A. (2020). Improving infant outcomes through implementation of a family integrated care bundle including a parent supporting mobile application. Archives of Disease in Childhood – Fetal and Neonatal Edition, 105(2), 172–177. https://doi.org/10.1136/archdischild-2018-316435<br><br>Bardach, S. H., Perry, A. N., Kapadia, N. S., Richards, K. E., Cogswell, L. K., & Hartman, T. K. (2022). Redesigning care to support earlier discharge from a neonatal intensive care unit: A design thinking informed pilot. BMJ Open Quality, 11(2), e001736. https://doi.org/10.1136/bmjoq-2021-001736<br><br>Boykova, M., & Kenner, C. (2012). Transition from hospital to home for parents of preterm infants. Journal of Perinatal & Neonatal Nursing, 26(1), 81–87. https://doi.org/10.1097/JPN.0b013e318243e948<br><br>Bozgan, J. et al. (2022). Gait Profile Score and Quality of Life in Children with Cerebral Palsy. Developmental Medicine & Child Neurology, 64(Suppl. 4), 80-138. https://doi.org/10.1111/dmcn.15356<br><br>Brassarola, H. G. M., Natarelli, T. R. P., & Fonseca, L. M. M. (2023). Use of the WhatsApp® group in the post-discharge follow-up of the premature infant: Implications for nursing care. Escola Anna Nery, 27, e20220205. https://doi.org/10.1590/2177-9465-EAN-2022-0205<br><br>Costa, B., Mozafarinia, M., Soehartojo, N. D., & Tedder, J. (2023). International, virtual resources and outreach to enhance care of the late preterm infant. International Journal of Birth and Parent Education, 10(4), 3–7.<br><br>Das, A., Cina, L., Mathew, A., Aziz, H., & Aly, H. (2020). Telemedicine, a tool for follow-up of infants discharged from the NICU? Experience from a pilot project. Journal of Perinatology, 40(6), 875–880. https://doi.org/10.1038/s41372-020-0593-5<br><br>Dathe, A. K., et al. (2024). Adaptation of follow-up visits for preterm and high-risk children for video consultations. Telemedicine and e-Health. Advance online publication. https://doi.org/10.1089/tmj.2023.0594<br><br>Ericson, J., Flacking, R., & Udo, C. (2017). Mothers' experiences of a telephone based breastfeeding support intervention after discharge from neonatal intensive care units: A mixed-method study. International Breastfeeding Journal, 12, 50. https://doi.org/10.1186/s13006-017-0142-9<br><br>Flores-Fenlon, N., Song, A. Y., Yeh, A., Gateau, K., Vanderbilt, D. L., Kipke, M., & Lakshmanan, A. (2019). Smartphones and text messaging are associated with higher parent quality of life scores and enrollment in early intervention after NICU discharge. Clinical Pediatrics, 58(8), 903–911. https://doi.org/10.1177/0009922819848080<br><br>Garfield, C. F., Kerrigan, E., Christie, R., Jackson, K. L., & Lee, Y. S. (2022). A mobile health intervention to support parenting self-efficacy in the neonatal intensive care unit from admission to home. The Journal of Pediatrics, 244, 92–100. https://doi.org/10.1016/j.jpeds.2022.01.004	35

Gholami, S., Farahani, A. S., Karahroudy, F. A., Moghadam, F., Boromandnia, N., & Mojen, L. K. (2022). The effect of telenursing on the rate of newborn readmission. Journal of Neonatal Nursing, 28(1), 26–30. https://doi.org/10.1016/j.jnn.2021.03.001

Gök, P. Y., & Efe, E. (2022). The effect of web-based preterm infant care training on mothers' self-confidence. Health Care for Women International, 43, 1080–1096. https://doi.org/10.1080/07399332.2022.2039150

Greene, M. M., Patra, K., Nelson, M. N., Silvestri, J. M., & Guthrie, L. M. (2020). Adaptation and acceptability of a digitally delivered intervention for parents of very low birth weight infants. Nursing Research, 69(5S Suppl. 1), S47–S56. https://doi.org/10.1097/NNR.0000000000000445

Guimarães, A. B., Marrero, L., Vidal, A. P., Oliveira, A. L., Menezes, E. G., Souza, A. A., & Teixeira, E. (2021). Desenvolvimento de tecnologia educacional para familiares sobre o banho de ofurô no domicílio para recém-nascidos. Enfermagem em Foco, 12(6), 1184–1188. https://doi.org/10.21675/2357-707X.2021.v12.n6.4861

Gustavsen, L. J., Le Marechal, F., & Tandberg, B. S. (2024). Observational study showed that using video consultations was a viable way of delivering an early discharge programme for preterm infants. Acta Paediatrica, 113(7), 1524–1530. https://doi.org/10.1111/apa.17250

Holm, K. G., Clemensen, J., Brødsgaard, A., Smith, A. C., Maastrup, R., & Zachariassen, G. (2019). Growth and breastfeeding of preterm infants receiving neonatal tele-homecare compared to hospital-based care. Journal of Neonatal-Perinatal Medicine, 12(3), 277–284. https://doi.org/10.3233/NPM-18143

Jang, E. H., & Ju, H. O. (2020). Effects of an infant care education program for mothers of late-preterm infants on parenting confidence, breastfeeding rates, and infants' growth and readmission rates. Child Health Nursing Research, 26(1), 11–22. https://doi.org/10.4094/chnr.2020.26.1.11

Jisha, M., Nandini, N., & Kishore, R. K. (2022). Routine CPR education of parents of preterm neonates backed by video demonstration: A retrospective study. Neonatology Today, 22(5), 3–8

Kavitha, K., Patil, M. M., & Sikandar, B. J. (2023). A digital-based follow-up program to improve maternal competence in preterm home care: A true-experimental study. Journal of Neonatal Nursing, 29(5), 750–754. https://doi.org/10.1016/j.jnn.2023.02.005

Koldoff, E. A. (2018). Transition to early intervention for infants with very low birth weight (Doctoral dissertation). University of Oklahoma Health Sciences Center

Lewis, J., Rowan, M., & Whitaker, A. (2022). Gait profile score and quality of life in children with cerebral palsy. Developmental Medicine & Child Neurology, 64(Suppl. 4), 80–138. https://doi.org/10.1111/dmcn.15356

Li, L., Li, Z., Wan, W., Li, J., Zhang, Y., Wang, C., & Wang, L. (2021). Management of follow-up with preterm infants during the outbreak in China. Frontiers in Pediatrics, 9, 637275. https://doi.org/10.3389/fped.2021.637275

Litt, J. S., Agni, M., Jacobi-Polishook, T., Melvin, P., McCormick, M. C., Stewart, J. E., & Belfort, M. B. (2018). The acceptability and feasibility of emailed parent questionnaires for medical and developmental surveillance after NICU discharge. Journal of Perinatology, 38(4), 392–401. https://doi.org/10.1038/s41372-017-0022-6

Luu, T. M., Xie, L. F., Peckre, P., Côté, S., Karsenti, T., Walker, C.-D., & Gosselin, J. (2017). Web-based intervention to teach developmentally supportive care to parents of preterm infants: Feasibility and acceptability study. JMIR Research Protocols, 6(11), e236. https://doi.org/10.2196/resprot.8289

Phagdol, T., Nayak, B. S., Lewis, L. E., & George, A. (2022). Designing a mobile health intervention for preterm home care: Application of conceptual framework. Public Health Nursing, 39(1), 296–302. https://doi.org/10.1111/phn.13020

Ra, J. S., & Lim, J. (2012). Development and evaluation of a video discharge education program focusing on mother-infant interaction for mothers of premature infants. Journal of Korean Academy of Nursing, 42(7), 936–946. https://doi.org/10.4040/jkan.2012.42.7.936

	Rasmussen, M. K., Clemensen, J., Zachariassen, G., Kidholm, K., Brødsgaard, A., Smith, A. C., & Holm, K. G. (2020). Cost analysis of neonatal tele-homecare for preterm infants compared to hospital-based care. Journal of Telemedicine and Telecare, 26(7–8), 474–481. https://doi.org/10.1177/1357633X19843753<br><br>Roman, L. A., Lindsay, J. K., Boger, R. P., DeWys, M., Beaumont, E. J., Jones, A. S., & Haas, B. (1995). Parent-to-parent support initiated in the neonatal intensive care unit. Research in Nursing & Health, 18(5), 385–394. https://doi.org/10.1002/nur.4770180504<br><br>Sarik, D. A., Matsuda, Y., Terrell, E. A., Sotolongo, E., Hernandez, M., Tena, F., & Lee, J. (2022). A telehealth nursing intervention to improve the transition from the neonatal intensive care unit to home for infants and caregivers: Preliminary evaluation. Journal of Pediatric Nursing, 67, 139–147. https://doi.org/10.1016/j.pedn.2022.09.003<br><br>Silva, R. M. M., Zilly, A., Nonose, E. R. S., Fonseca, L. M. M., & Mello, D. F. (2020). Care opportunities for premature infants: Home visits and telephone support. Revista Latino-Americana de Enfermagem, 28, e3308. https://doi.org/10.1590/1518-8345.3520.3308<br><br>Suir, I., Oosterhaven, J., Boonzaaijer, M., Nuysink, J., & Jongmans, M. (2022). The AIMS home-video method: Parental experiences and appraisal for use in neonatal follow-up clinics. BMC Pediatrics, 22, 338. https://doi.org/10.1186/s12887-022-03398-9<br><br>Zhang, J., Li, Y., Zhu, L., Shang, Y., & Yan, Q. (2024). The effectiveness of an online breastfeeding education and support program on mothers of preterm infants: A quasi-experimental study. Midwifery, 130, 103924. https://doi.org/10.1016/j.midw.2024.103924	
**Language**<br>*Articles in languages without available translations (e.g., Korean)*	Khodaparast, M., Rahani, T., Sadeghi, T., Boskabadi, H., & Yavari, M. (2018). The effect of training through telenursing and multimedia on the success of lactation in preterm infants after discharge. Journal of North Khorasan University of Medical Sciences, 10(2), 13–20. https://doi.org/10.52547/nkums.10.2.13<br><br>Lee, N.-Y., & Kim, Y.-H. (2008). Development and evaluation of an e-learning program for mothers of premature infants. Journal of Korean Academy of Nursing, 38(1), 152–160. https://doi.org/10.4040/jkan.2008.38.1.152	2
