# Supplementary material for: Effectiveness of eHealth interventions for parents of preterm infants during transition from neonatal intensive care unit-to-home: A systematic review and meta-analysis
Source: Int J Nurs Stud Adv. 2026 Sep 21;11:100684. doi: 10.1016/j.ijnsa.2026.100684 (PMC13653947; doi:10.1016/j.ijnsa.2026.100684)
Supplement: Supplementary file 3 [file mmc5.pdf]

Supplementary file S3 - Characteristics of included studies (n=17)

Author (Year, Country)	Early Intervention Programme	Population / Context	Mode of Delivery<br>I -Individual<br>G = Group	Delivered by	Intervention Components<br>(1) Psychosocial Support<br>(2) Parent Education<br>(3) Infant Support/Therapy	Frequency / Duration	Parental Outcomes Measured	Infant Outcomes Measured	Key Findings	Level of Evidence*
**Asghari et al. (2021, Iran)**	Telenursing Program via Telegram application (*mHealth*)	120 mothers of preterm infants following NICU discharge	I	Researcher / Nurse	(1) Telephone counselling and emotional support<br>(2) Structured weekly Telegram sessions on breastfeeding, hygiene, feeding difficulties, infant massage, and sleep regulation	Weekly sessions × 4 weeks (~30 min)<br>*Timing: post-discharge only*	Parental stress *(PSS – Berry & Jones)*	—	↓ Maternal stress immediately and 4 weeks post-intervention (p < 0.001 within-group; between-group p < 0.05)	Low
**Bahmanpour et al. (2023, Iran)**	Telenursing Training Program via Instagram (*mHealth*)	70 mothers of preterm infants following NICU discharge	I	Researcher / Nurse	(1) Continuous emotional support via social media<br>(2) Daily parent education through posts and messages on infant care	Daily sessions over 4 weeks<br>*Timing: post-discharge only*	Maternal hope (*HS*)<br>Parental self-efficacy (*PMPS-Q*)	—	↑ Maternal hope and ↑ parental self-efficacy immediately and 4 weeks post-intervention (within-group p < 0.001; between-group p < 0.01)	Moderate
**Colditz et al. (2019, Australia)**	Baby Triple P for Preterm Infants (*Hybrid – group + telephone*)	323 families of infants born <32 weeks' GA during NICU hospitalization and community follow-up	G + I	Psychologists (Trained facilitators)	(1) Psychosocial support focusing on coping skills and partner communication<br>(2) Parent education on effective parenting strategies and recognizing infant needs<br>(3) Parent-mediated developmental support – guidance on creating a nurturing home environment to promote early child development	8 sessions:<br><br>4 group hospital sessions (2 h) + 4 weekly 30-min telephone consultations starting at 2 weeks' CA<br>*Timing: pre- and post-discharge*		Cognitive, language, and motor development *(Bayley-III)*<br>Behavioural outcomes *(ITSEA, FOS)*<br>Mother–child interaction *(CSBS DP)*	↑ Cognitive (Δ = 3.5, p = 0.04) and ↑ motor development (Δ = 5.5, p < 0.001) at 2 years' CA; borderline ↑ language (p = 0.07)	High
**Ericson et al. (2018, Sweden)**	Proactive telephone support to beastfeeding (*Telehealth – telephone*)	493 mothers of preterm infants (<37 weeks' GA) in transition from NICU to home	I	Nurses (Breastfeeding Support Team)	(1) Person-centred daily telephone calls providing emotional support<br>(2) Parent education on breastfeeding, bonding, and maternal well-being	Daily calls for 14 days (including weekends)<br>*Timing: post-discharge only*	Exclusive breastfeeding at 8 weeks *(MBES)*<br>Maternal satisfaction with breastfeeding *(MBES)*<br>Parental stress *(PSS – Swedish)*		↓ Parental stress (p < 0.05)<br>No significant differences between groups in exclusive breastfeeding, maternal satisfaction with breastfeeding, attachment, or quality of life	High
**Fratantoni et al. (2022, USA)**	Giving Parents Support with Peer Navigators (*Telehealth/peer support*)	300 families of preterm infants during the transition from NICU to home	I	Peer Navigators / Research team (NICU)	(1) Peer-to-peer emotional support and empowerment before and after discharge<br>(2) Guidance on communication skills, resource navigation, and coordination with healthcare and community services	Initial contact pre-discharge; follow-up within 2 weeks post-discharge and monthly peer support for 12 months<br>*Timing: pre- and post-discharge*	Parental stress *(PSS-10)*<br>Depression *(CES-D-10)*<br>Anxiety *(GAD)*<br>Parental self-efficacy *(PMPS-E)*	Infant hospitalizations and emergency department visits *(parent-reported)*<br>Developmental outcomes (*Bayley-III*)	No significant differences betweengroup; overall improvement in parental mental health in both groups; intervention feasible and acceptable	High
**Garfield et al. (2016, USA)**	*NICU-2-Home* smartphone application (*mHealth app*)	90 parents of very-low-birth-weight (VLBW) infants during	I	Research team (NICU)/ Developers	(1) Emotional tracking and partner mood feedback *(Mood Tracker)*<br>(2) Multimedia education and discharge preparedness checklist *(Education Center, Passport-2-Home)*	Continuous app use during the 4-week NICU-to-home transition	Parenting self-efficacy *(PSOC)*<br>Discharge preparedness *(PGDQ)*	Length of hospital stay *(LOS)*	↑ Parenting self-efficacy among high-use participants (p < 0.001)<br>↑ Discharge preparedness (p < 0.001)	Moderate

		the NICU-to-home transition			(3) Parent-mediated infant monitoring and shared record keeping *(Baby Connect, Notes)*	*Timing: pre- and post-discharge*			Trend toward shorter hospital stay (*p* = 0.085)	
**Gund et al. (2013, Sweden)**	eHealth home care (web + Skype) (*Telehealth - web-based*)	34 families of preterm infants receiving neonatal home care after hospital discharge	I	Neonatal home care nurses	(1) Emotional reassurance and communication with nurses via Skype video calls<br>(2) Remote guidance and web-based information<br>(3) Parent-mediated infant monitoring via web platform and video contacts	Variable web/video contacts during home care over ~2–3 weeks<br>*Timing: post-discharge only*	Parental satisfaction and confidence in care (*Likert-based Satisfaction Questionnaire*)	—	↑ Parental confidence and satisfaction<br>↓ Home visits compared with standard care<br>*p*-values not reported (feasibility study)	Low
**Haemmerli et al. (2024,Switzerland)**	Transition to Home (TtH) model (*Hybrid – APN-led program*)	45 parent couples of preterm infants enrolled in a structured transition-to-home program	I	Advanced Practice Nurses (APNs) with psychologists	(1) Individualized emotional and psychological support by APNs in collaboration with psychologists<br>(2) Tailored guidance to strengthen caregiving competences, mental health literacy, and self-efficacy	Individualised support over 6 months post-discharge<br>*Timing: post-discharge only*	Depression *(CES-D)*<br>Anxiety *(STAI)*<br>Post-traumatic stress *(PCL-5)*<br>Parenting self-efficacy *(TOPSE)*	—	↓ Maternal depression at T2 (*p* = 0.042) and T5 (*p* = 0.027)<br>↓ Paternal state anxiety at T2 (*p* = 0.016)<br>No significant effects on parenting stress or self-efficacy<br>Feasibility confirmed	High
**Hägi-Pedersen et al. (2022, Denmark)**	Early In-Home Care Program (Video Consultations) (*Telehealth – video*)	160 families of preterm infants receiving early in-home neonatal care	I	Neonatal nurses (IBCLC-certified)	(1) Emotional counselling and reassurance via video consultations<br>(2) Breastfeeding guidance, infant care advice, and support for early in-home transition	2–3 planned video consultations per week over ~18 days<br>*Timing: post-discharge only*	Parenting confidence *(KPCS)*<br>Exclusive breastfeeding at discharge and 1 month *(MBES)*<br>Mother–infant interaction *(MABISC)*		No significant differences in exclusive breastfeeding at discharge or 1 month<br>Parenting confidence (KPCS) and mother–infant interaction (MABISC) similar across groups	High
**Huang et al. (2024, China)**	Online Family-focused Parenting Support (Tencent Meeting) (*Web-based video*)	89 families of preterm infants during early home transition	G + I	Nurses via online platform	(1) Emotional and motivational encouragement in group and individual online sessions<br>(2) Structured teaching using videos, slides, and manikin demonstrations<br>(3) Parent-mediated infant care training reinforced through follow-up calls	2 group + 3 individual online sessions + 2 follow-up calls over 3 months<br>*Timing: post-discharge only*	Parenting competence *(C-PSOC)*<br>Depression *(EPDS)*<br>Social support *(SSRS)*	Infant growth *(weight, length)*	↑ Parenting competence and infant care competence (*p* < 0.001 over time; between-group *p* < 0.05)<br>↓ Depressive symptoms (*p* < 0.001)<br>No significant differences in infant growth	High
**Jafarzadeh et al. (2019, Iran)**	Telenursing Program using an Interactive Voice Response (IVR) system (*mHealth – IVR*)	50 mothers of preterm infants during NICU stay and early post-discharge period	I	Nurse / Researcher	(1) Telephone counselling and emotional reassurance during NICU stay and after discharge<br>(2) Structured educational content via IVR on bonding, neonatal care, and breastfeeding	Brief IVR/telephone contacts from NICU admission to 1 week post-discharge<br>*Timing: pre- and early post-discharge*	Parental stress *(PSS-NICU)*<br>Maternal attachment *(MPA)*	—	↓ Parental stress (PSS-NICU) and ↑ maternal attachment (MPA) (*p* < 0.05)	Moderate
**Mobbs et al. (2022, Australia)**	*PreEMPT* - Preterm infant Early intervention for Movement and Participation Trial (*Hybrid – telehealth + in-*	16 preterm infants (≤34 weeks' GA) and their parents	I	Physiotherapists	(2) Participation-focused coaching to enhance parental skills and confidence in supporting infant motor development<br>(3) Early physiotherapy program combining clinic-based and telehealth sessions to promote motor performance and participation	Weekly tele-/in-person sessions over 14 weeks<br>*Timing: post-discharge only*	Parenting self-efficacy *(PSOC)*<br>Anxiety and depression *(HADS)*<br>Satisfaction *(study-specific survey)*	Motor performance *(TIMP, AIMS, NSMDA)*<br>Development *(Bayley-III)*	↑ Parenting self-efficacy (PSOC) at 4 months' corrected age (*p* = 0.02)<br>No significant differences in anxiety (*p* = 0.45) or depression (*p* = 0.64)<br>Intervention feasible	High

	person physiotherapy)									
**Mohammadian et al. (2021, Iran)**	Continuous Supportive Telephone Breastfeeding Counselling (*Telehealth – telephone*)	65 mothers of late preterm infants (34–37 weeks' GA) after hospital discharge	I	Nurse counsellor	(1) Continuous emotional and motivational support via daily telephone contact<br>(2) Structured breastfeeding counselling on technique, problem-solving, and maternal confidence	Daily counselling for 14 days with follow-up to 4 months<br>*Timing: post-discharge only*	Breastfeeding self-efficacy *(BSES)*	—	↑ Breastfeeding self-efficacy sustained up to 4 months post-discharge ($p < 0.001$)	Moderate
**Neyestani et al. (2017, Iran)**	Structured discharge education + telephone follow-up (*Hybrid – in-person + telephone*)	60 mothers of preterm infants (30–35 weeks' GA)	I	Researchers / Nurses	(1) Post-discharge telephone follow-up providing reassurance and emotional support<br>(2) Four structured in-hospital training sessions on premature infant care, breastfeeding, hygiene, and developmental screening	4 pre-discharge sessions (twice weekly) + 4 weeks of telephone follow-up<br>*Timing: pre- and post-discharge*	Quality of life *(WHOQOL-BREF)*<br>Depression *(EPDS)*<br>Social support *(MSPSS)*	—	↑ Maternal quality of life at discharge and at 4 weeks post-intervention ($p < 0.001$)	High
**Phagdol et al. (2023, India)**	mHealth application for preterm home care (*mHealth app*)	160 mothers of preterm infants after NICU discharge	I	Researcher / App developer	(1) Remote encouragement and connection with the healthcare team via app<br>(2) Interactive modules with videos and educational resources on KMC, feeding, hygiene, growth, and danger signs<br>(3) Parent-mediated infant care through practical caregiving instructions supported by app guidance	Continuous app use throughout a 12-month follow-up period<br>*Timing: post-discharge only*	Knowledge of preterm home care *(Structured Knowledge Questionnaire)*<br>Acceptability/usability ratings	—	↑ Knowledge scores regarding preterm home care ($p < 0.001$)<br>High acceptability and usability of the mHealth app	Moderate
**Robinson et al. (2016, Sweden)**	Telemedicine home follow-up (web + videocalls) (*Telehealth + web-based*)	139 families of preterm infants	I	Neonatal nurses	(1) Nurse-led video consultations providing reassurance, guidance, and emotional support<br>(2) Daily web-based questionnaires with individualised nurse feedback<br>(3) Parent-mediated infant care through practical caregiving instructions via telemedicine platform	Approximately 10 web forms and 1–5 nurse-led video calls over a 3-week home care period<br>*Timing: post-discharge only*	Parental satisfaction and confidence *(study-specific survey)*<br>Acceptability	Hospital and emergency visits during home care follow-up	↑ Parental confidence and satisfaction<br>↓ Number of hospital visits compared with standard care<br>Feasibility confirmed; *p*-values not reported	Moderate
**Yan et al. (2022, China)**	WeChat-based remote follow-up management (*mHealth – WeChat platform*)	150 parents of preterm infants	I	Medical staff via WeChat group	(1) Online peer and professional support via WeChat group with daily communication and emotional reassurance<br>(2) Educational care modules and live Q&A sessions on feeding, care routines, and complication management<br>(3) Parent-mediated infant monitoring through remote reporting of infant condition	Daily WeChat interaction for 4 weeks after discharge<br>*Timing: post-discharge only*	Care burden *(ZBI, FCTI)*<br>Anxiety *(SAS)*<br>Depression *(SDS)*<br>Satisfaction	—	↓ Parental burden (ZBI, FCTI), ↓ anxiety (SAS), and ↓ depression (SDS) ($p < 0.05$)<br>↑ Parental satisfaction with remote support	High

Abbreviations: GA, gestational age; NICU, Neonatal Intensive Care Unit; CA, corrected age; VLBW, very-low-birth-weight; ED, emergency department; PSS - Berry & Jones, Parental Stress Scale (Berry & Jones); HS, Hope Scale; PMPS-Q/E, Perceived Maternal Parenting Self-Efficacy Questionnaire/Scale; Bayley-III, *Bayley Scales of Infant and Toddler Development* (3rd ed.); ITSEA, *Infant-Toddler Social and Emotional Assessment*; FOS, *Family Observation Schedule*; CSBS DP, *Communication and Symbolic Behavior Scales Developmental Profile*; MBES, *Maternal Breastfeeding Evaluation Scale*; PSOC, *Parenting Sense of Competence Scale*; PGDQ, *Press-Ganey Discharge Questionnaire*; CES-D, *Center for Epidemiologic Studies Depression Scale*; STAI, *State–Trait Anxiety Inventory*; PCL-5, *PTSD Checklist for DSM-5*; TOPSE, *Tool to Measure Parenting Self-Efficacy*; KPCS, *Karitane Parenting Confidence Scale*; MABISC, *Mother and Baby Interaction Scale*; C-PSOC, Chinese version of PSOC; EPDS, *Edinburgh Postnatal Depression Scale*; SSRS, *Social Support Rating Scale*; PSS-NICU, *Parental Stressor Scale: Neonatal Intensive Care Unit*; MPA, *Maternal Postnatal Attachment Questionnaire*; HADS, *Hospital Anxiety and Depression Scale*; TIMP, *Test of Infant Motor Performance*; AIMS, *Alberta Infant Motor Scale*; NSMDA, *Neurosensory Motor Developmental Assessment*; WHOQOL-BREF, *World Health Organization Quality of Life – Brief*; MSPSS, *Multidimensional Scale of Perceived Social Support*; BSES, *Breastfeeding Self-Efficacy Scale*; ZBI, *Zarit Burden Interview*; FCTI, *Family Caregiver Task Inventory*; SAS, *Self-Rating Anxiety Scale*; SDS, *Self-Rating Depression Scale.*
**Level of evidence classified according to the total number of "Yes" judgments in the JBI critical appraisal checklist for randomized controlled trials (Ames et al., 2023): High (≥10/13 "Yes"), Moderate (9/13 "Yes"), Low (≤8/13 "Yes").*
