# Supplementary material for: Effectiveness of eHealth interventions for parents of preterm infants during transition from neonatal intensive care unit-to-home: A systematic review and meta-analysis
Source: Int J Nurs Stud Adv. 2026 Sep 21;11:100684. doi: 10.1016/j.ijnsa.2026.100684 (PMC13653947; doi:10.1016/j.ijnsa.2026.100684)

Supplementary File S4. Forest Plots

Forest plot of the effect of eHealth early intervention programmes on **parental anxiety** at 3 and 6 months after neonatal intensive care unit discharge.

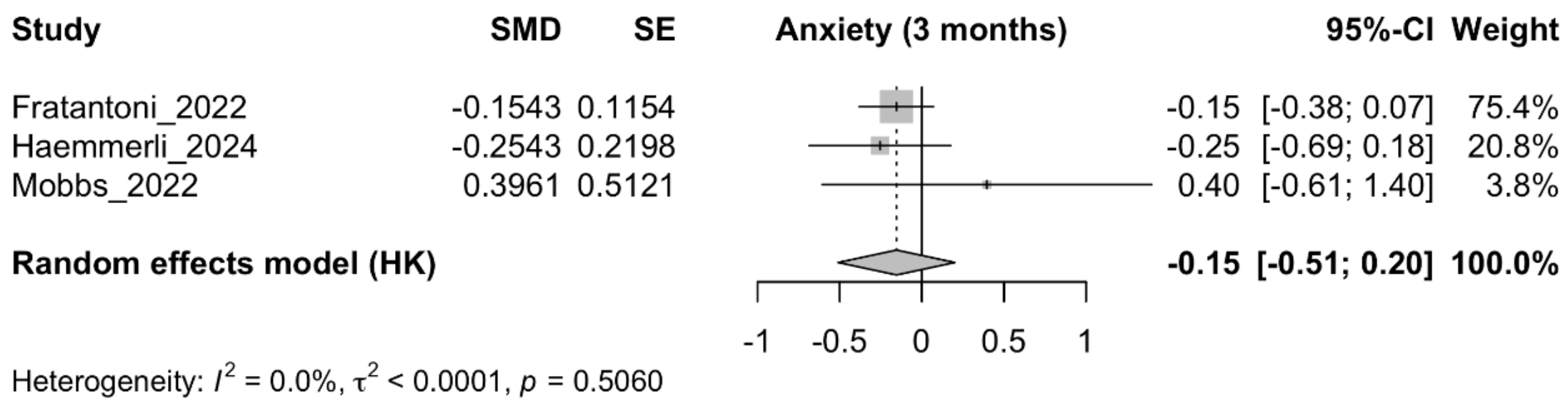

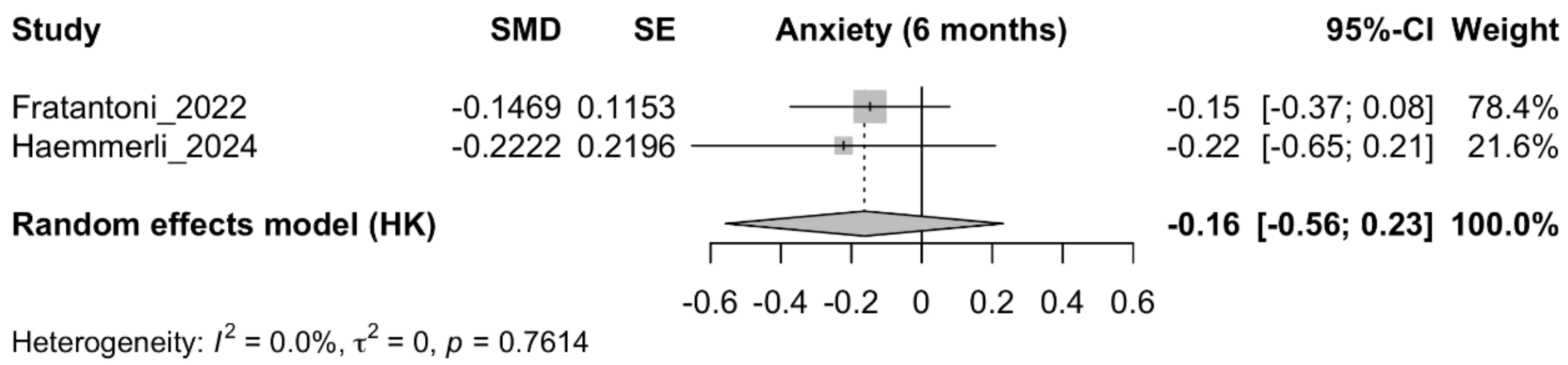

Forest plot of the effect of eHealth early intervention programmes on **parental depressive symptoms** at 1,3 and 6 months after neonatal intensive care unit discharge.

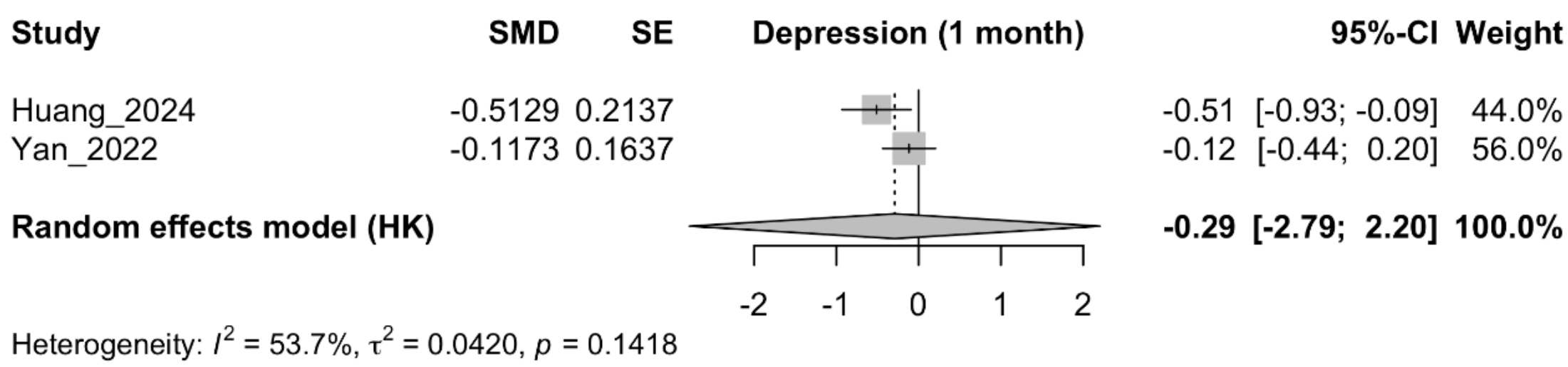

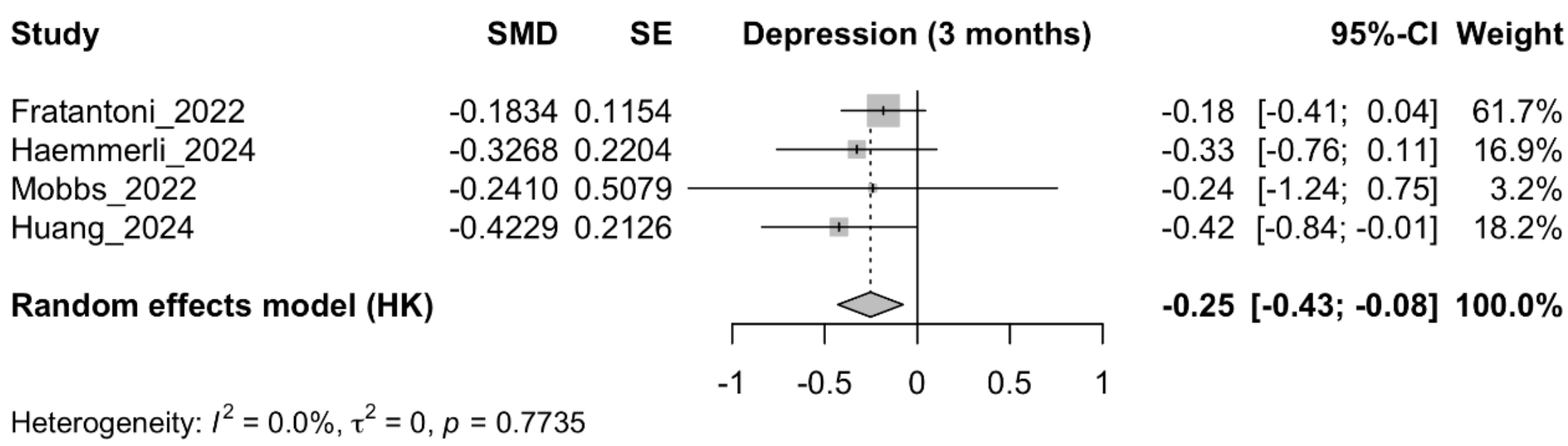

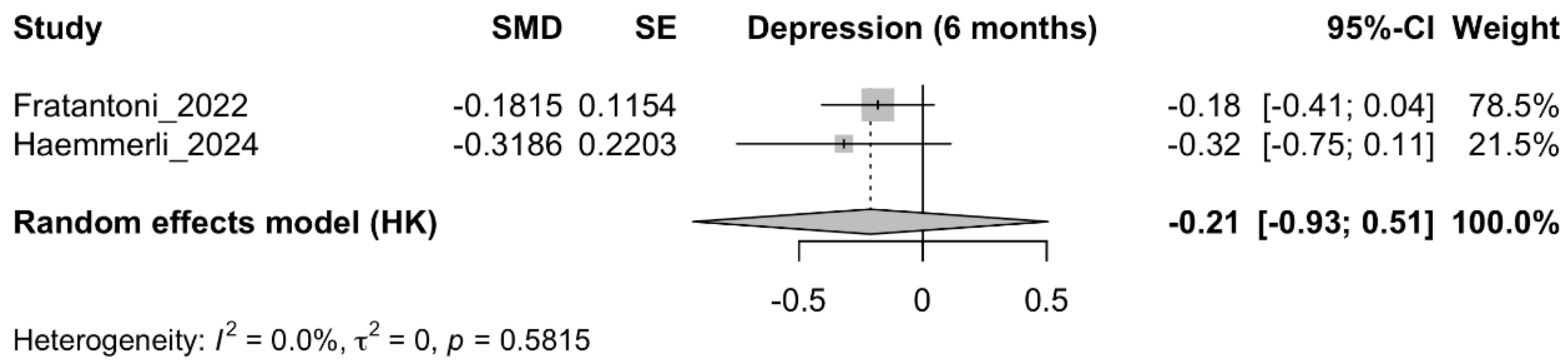

Forest plot of the effect of eHealth early intervention programmes on **self-efficacy** at 1,3 and 6 months after neonatal intensive care unit discharge.

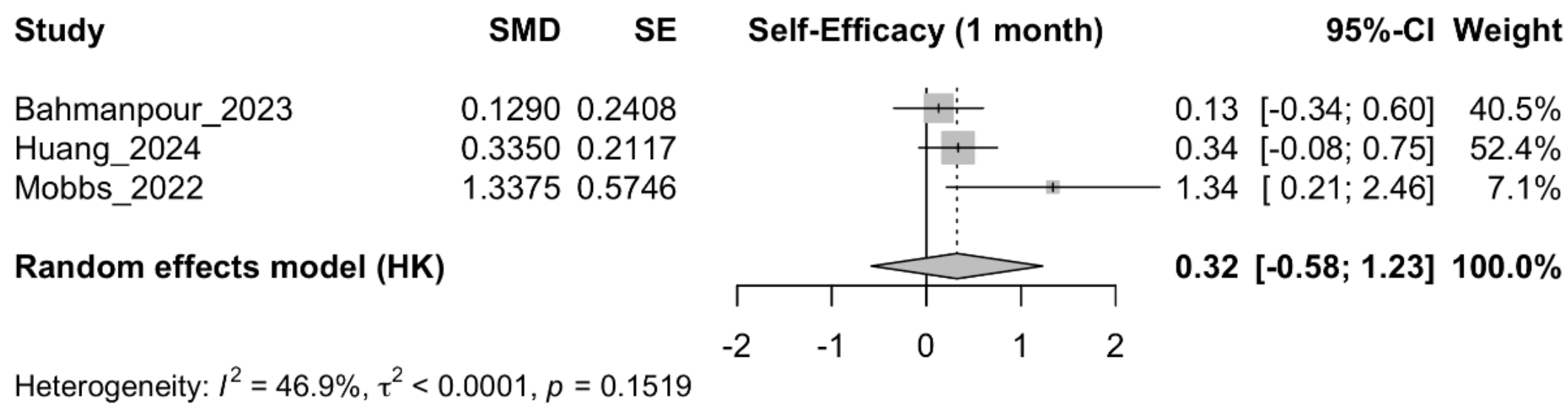

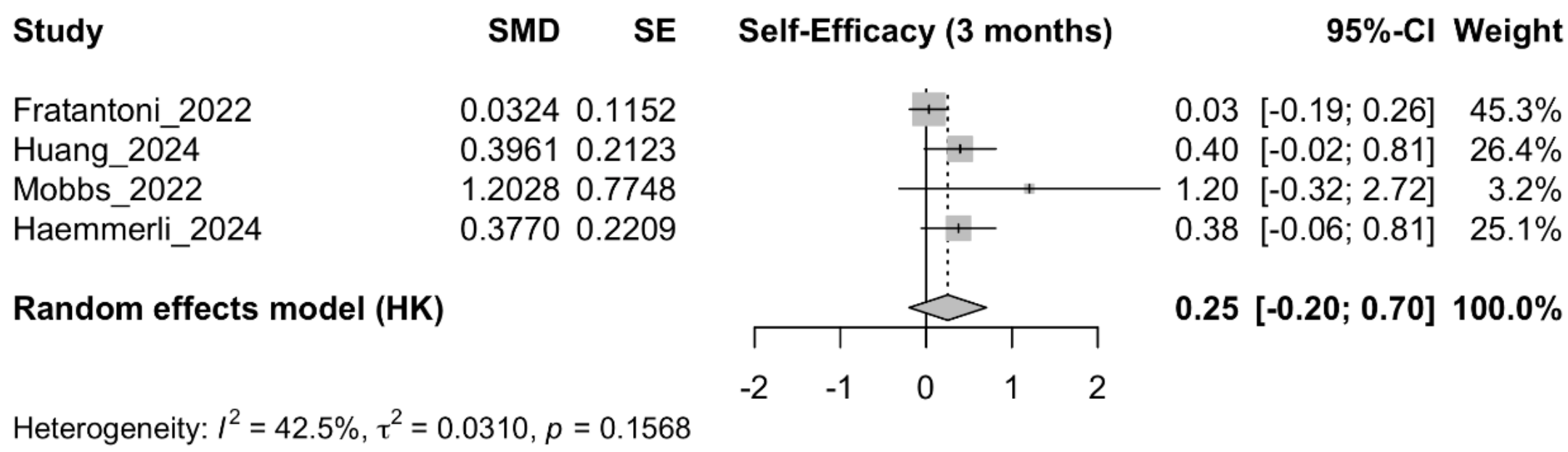

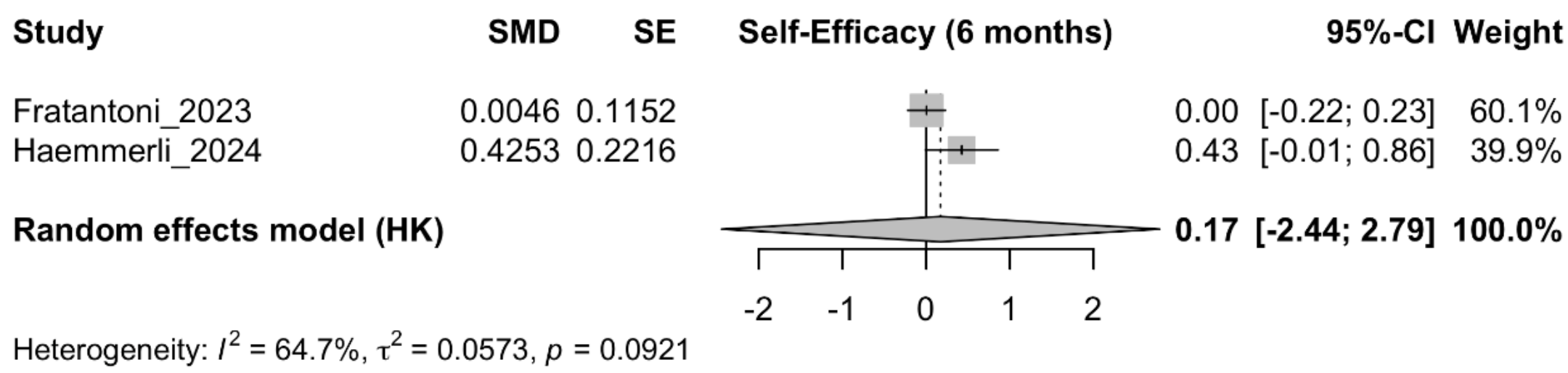

Forest plot of the effect of eHealth early intervention programmes on **parental stress** at 3 and 6 months after neonatal intensive care unit discharge.

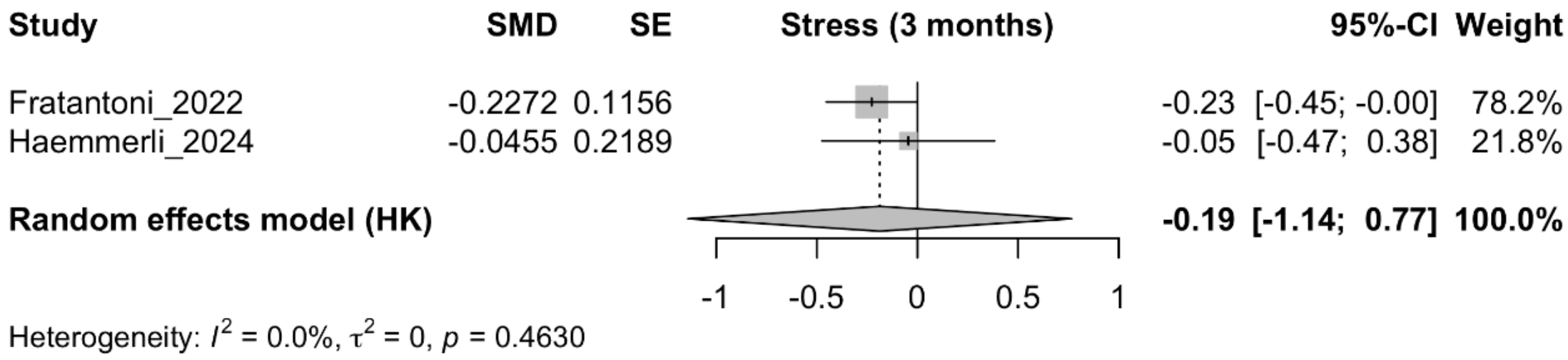

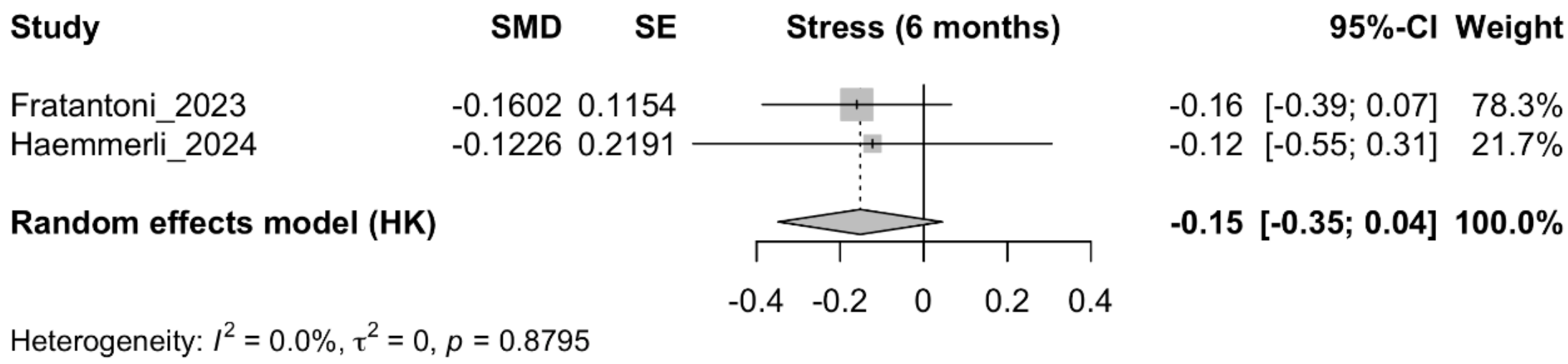

Supplement: Supplementary file 4 [file mmc6.pdf]
