# Supplementary material for: Effectiveness of eHealth interventions for parents of preterm infants during transition from neonatal intensive care unit-to-home: A systematic review and meta-analysis
Source: Int J Nurs Stud Adv. 2026 Sep 21;11:100684. doi: 10.1016/j.ijnsa.2026.100684 (PMC13653947; doi:10.1016/j.ijnsa.2026.100684)
Supplement: Supplementary file 5 [file mmc2.docx]

The PRISMA 2020 reporting checklist

Effectiveness of eHealth interventions for parents of preterm infants during the NICU-to-home transition: A systematic review and meta-analysis

How to use this reporting checklist

This reporting checklist allows authors to demonstrate that their manuscripts adhere to the [PRISMA 2020 reporting guideline](https:/resources.equator-network.org/reporting-guidelines/prisma/index.html).

If you have not used a reporting guideline before, read about [how and why to use them](https:/resources.equator-network.org/about/reporting-guidelines.html) and check whether PRISMA 2020 is the [most applicable reporting guideline](https:/resources.equator-network.org/reporting-guidelines/prisma/index.html?#applicability) for your work.

Reporting guidelines are most useful when used early in research. When writing a manuscript or application, consider using the [full guidance](https:/resources.equator-network.org/reporting-guidelines/prisma/index.html) where you’ll find explanations and examples for each item.

After writing, demonstrate adherence by completing this checklist:

1. Specify where each item is described (see Note 1).
2. Cite this checklist (See Note 2).
3. Include your completed checklist as a supplement when submitting to a journal so that future readers can use it to find information.

|  | Item Description | Location (or reason for not reporting) |
| --- | --- | --- |
| **Title and Abstract** |  |  |
| [1. Title](https:/resources.equator-network.org/reporting-guidelines/prisma/items/title.html?utm_source=prisma&utm_medium=checklist&utm_campaign=1_1) | Identify the report as a systematic review. | Title page |
| [2. Abstract](https:/resources.equator-network.org/reporting-guidelines/prisma/items/abstract.html?utm_source=prisma&utm_medium=checklist&utm_campaign=1_1) | Include all items from the *PRISMA 2020 for Abstracts* checklist. | Abstract (p.1) |
| **Introduction** |  |  |
| [3. Rationale](https:/resources.equator-network.org/reporting-guidelines/prisma/items/rationale.html?utm_source=prisma&utm_medium=checklist&utm_campaign=1_1) | Describe the rationale for the review in the context of existing knowledge. | Introduction (p.3–7) |
| [4. Objectives](https:/resources.equator-network.org/reporting-guidelines/prisma/items/objectives.html?utm_source=prisma&utm_medium=checklist&utm_campaign=1_1) | Provide an explicit statement of the objective(s) or question(s) the review addresses. | Introduction – final paragraph (p.7) |
| **Methods** |  |  |
| [5. Eligibility criteria](https:/resources.equator-network.org/reporting-guidelines/prisma/items/eligibility-criteria.html?utm_source=prisma&utm_medium=checklist&utm_campaign=1_1) | Specify the inclusion and exclusion criteria for the review and how studies were grouped for the syntheses. | Methods – Eligibility criteria (p.8) and Table 1 |
| [6. Information sources](https:/resources.equator-network.org/reporting-guidelines/prisma/items/information-sources.html?utm_source=prisma&utm_medium=checklist&utm_campaign=1_1) | Specify all databases, registers, websites, organisations, reference lists and other sources searched or consulted to identify studies. Specify the date when each source was last searched or consulted. | Methods – Search methods (p.9) |
| [7. Search](https:/resources.equator-network.org/reporting-guidelines/prisma/items/search.html?utm_source=prisma&utm_medium=checklist&utm_campaign=1_1) | Present the full search strategies for all databases, registers and websites, including any filters and limits used. | Methods – Search methods (p.9) and Supplementary file S1 |
| [8. Selection Process](https:/resources.equator-network.org/reporting-guidelines/prisma/items/selection-process.html?utm_source=prisma&utm_medium=checklist&utm_campaign=1_1) | Specify the methods used to decide whether a study met the inclusion criteria of the review, including how many reviewers screened each record and each report retrieved, whether they worked independently, and, if applicable, details of automation tools used in the process. | Methods – Study selection (p.9-10) and Supplementary file S2 |
| [9. Data collection process](https:/resources.equator-network.org/reporting-guidelines/prisma/items/data-collection-process.html?utm_source=prisma&utm_medium=checklist&utm_campaign=1_1) | Specify the methods used to collect data from reports, including how many reviewers collected data from each report, whether they worked independently, any processes for obtaining or confirming data from study investigators, and if applicable, details of automation tools used in the process. | Methods – Data extraction (p.10-11) and Supplementary file S3 |
| 10. Data Items |  |  |
| [10a. Outcomes](https:/resources.equator-network.org/reporting-guidelines/prisma/items/data-items-outcomes.html?utm_source=prisma&utm_medium=checklist&utm_campaign=1_1) | List and define all outcomes for which data were sought. Specify whether all results that were compatible with each outcome domain in each study were sought (e.g. for all measures, time points, analyses), and if not, the methods used to decide which results to collect. | Methods – Data extraction (p.10-11) and Supplementary file S3 |
| [10b. Other Variables](https:/resources.equator-network.org/reporting-guidelines/prisma/items/data-items-other-variables.html?utm_source=prisma&utm_medium=checklist&utm_campaign=1_1) | List and define all other variables for which data were sought (e.g. participant and intervention characteristics, funding sources). Describe any assumptions made about any missing or unclear information. | Methods – Data extraction (p.10-11) and Supplementary file S3 |
| [11. Risk of bias in individual studies](https:/resources.equator-network.org/reporting-guidelines/prisma/items/risk-of-bias-in-individual-studies.html?utm_source=prisma&utm_medium=checklist&utm_campaign=1_1) | Specify the methods used to assess risk of bias in the included studies, including details of the tool(s) used, how many reviewers assessed each study and whether they worked independently, and if applicable, details of automation tools used in the process. | Methods – Methodological quality appraisal (p.10) |
| [12. Effect measures](https:/resources.equator-network.org/reporting-guidelines/prisma/items/effect-measures.html?utm_source=prisma&utm_medium=checklist&utm_campaign=1_1) | Specify for each outcome the effect measure(s) (e.g. risk ratio, mean difference) used in the synthesis or presentation of results. | Methods – Data synthesis and analysis (pp.10–11) |
| 13. Synthesis Methods |  |  |
| [13a. Deciding which studies were eligible for each synthesis](https:/resources.equator-network.org/reporting-guidelines/prisma/items/synthesis-methods-eligibility.html?utm_source=prisma&utm_medium=checklist&utm_campaign=1_1) | Describe the processes used to decide which studies were eligible for each synthesis (such as tabulating the study intervention characteristics and comparing against the planned groups for each synthesis described in item 5. | Methods – Data extraction (p.10-11) and Supplementary file S3  Methods – Data synthesis and analysis (p.11-12) |
| [13b. Data preparation methods](https:/resources.equator-network.org/reporting-guidelines/prisma/items/synthesis-methods-data-preparation.html?utm_source=prisma&utm_medium=checklist&utm_campaign=1_1) | Describe any methods required to prepare the data for presentation or synthesis, such as handling of missing summary statistics, or data conversions. | Methods – Data extraction (p.10-11) and Supplementary file S3  Methods – Data synthesis and analysis (p.11-12) |
| [13c. Methods for tabulating or displaying results](https:/resources.equator-network.org/reporting-guidelines/prisma/items/synthesis-methods-tabulating-or-displaying-results.html?utm_source=prisma&utm_medium=checklist&utm_campaign=1_1) | Describe any methods used to tabulate or visually display results of individual studies and syntheses. | Methods  Data extraction (p.10-11) and Supplementary file S3  Data synthesis and analysis (p.11-12)  Certainty of evidence (p.12) |
| [13d. Synthesis methods](https:/resources.equator-network.org/reporting-guidelines/prisma/items/synthesis-methods-synthesis-methods.html?utm_source=prisma&utm_medium=checklist&utm_campaign=1_1) | Describe any methods used to synthesize results and provide a rationale for the choice(s). If meta-analysis was performed, describe the model(s), method(s) to identify the presence and extent of statistical heterogeneity, and software package(s) used. | Methods  Data synthesis and analysis (p.11-12)  Certainty of evidence (p.12) |
| [13e. Methods for exploring heterogeneity](https:/resources.equator-network.org/reporting-guidelines/prisma/items/synthesis-methods-exploring-heterogeneity.html?utm_source=prisma&utm_medium=checklist&utm_campaign=1_1) | Describe any methods used to explore possible causes of heterogeneity among study results (e.g. subgroup analysis, meta-regression). | Methods  Data synthesis and analysis (p.11-12)  Certainty of evidence (p.12) |
| [13f. Sensitivity analyses](https:/resources.equator-network.org/reporting-guidelines/prisma/items/synthesis-methods-sensitivity-analyses.html?utm_source=prisma&utm_medium=checklist&utm_campaign=1_1) | Describe any sensitivity analyses conducted to assess robustness of the synthesized results. | Not performed |
| [14. Reporting bias assessment](https:/resources.equator-network.org/reporting-guidelines/prisma/items/reporting-bias-assessment.html?utm_source=prisma&utm_medium=checklist&utm_campaign=1_1) | Describe any methods used to assess risk of bias due to missing results in a synthesis (arising from reporting biases). | Methods  Methodological quality appraisal (p.10)  Certainty of evidence (p.12) |
| [15. Certainty assessment](https:/resources.equator-network.org/reporting-guidelines/prisma/items/certainty-assessment.html?utm_source=prisma&utm_medium=checklist&utm_campaign=1_1) | Describe any methods used to assess certainty (or confidence) in the body of evidence for an outcome. | Methods – Certainty of evidence (p.12) |
| **Results** |  |  |
| 16. Study Selection |  |  |
| [16a. Results of the search and selection process](https:/resources.equator-network.org/reporting-guidelines/prisma/items/study-selection-search-results.html?utm_source=prisma&utm_medium=checklist&utm_campaign=1_1) | Describe the results of the search and selection process, from the number of records identified in the search to the number of studies included in the review, ideally using a flow diagram. | Results – Study inclusion (p.12) and Figure 1 |
| [16b. Excluded studies](https:/resources.equator-network.org/reporting-guidelines/prisma/items/study-selection-excluded-studies.html?utm_source=prisma&utm_medium=checklist&utm_campaign=1_1) | Cite studies that might appear to meet the inclusion criteria, but which were excluded, and explain why they were excluded. | Supplementary file S2 |
| [17. Study characteristics](https:/resources.equator-network.org/reporting-guidelines/prisma/items/study-characteristics.html?utm_source=prisma&utm_medium=checklist&utm_campaign=1_1) | Cite each included study and present its characteristics. | Results – Characteristics of included studies (p.13–14); Supplementary file S3 |
| [18. Risk of bias in studies](https:/resources.equator-network.org/reporting-guidelines/prisma/items/risk-of-bias-in-studies.html?utm_source=prisma&utm_medium=checklist&utm_campaign=1_1) | Present assessments of risk of bias for each included study. | Results – Methodological quality (p.13) and Table 2 |
| [19. Results of individual studies](https:/resources.equator-network.org/reporting-guidelines/prisma/items/results-of-individual-studies.html?utm_source=prisma&utm_medium=checklist&utm_campaign=1_1) | For all outcomes, present, for each study: (a) summary statistics for each group (where appropriate) and (b) an effect estimate and its precision (e.g. confidence/credible interval), ideally using structured tables or plots. | Results – Review findings (pp.16–18) and Supplementary file S4 |
| 20. Results of Synthesis |  |  |
| [20a. Summary of studies](https:/resources.equator-network.org/reporting-guidelines/prisma/items/results-of-syntheses-summary-of-studies.html?utm_source=prisma&utm_medium=checklist&utm_campaign=1_1) | For each synthesis, briefly summarise the characteristics and risk of bias among contributing studies. | Results  Study inclusion Methodological quality  Review findings (p.16–18) and Supplementary file S2, Figure 1, Table 2, Table 3Supplementary files S3 and S4 |
| [20b. Statistical results](https:/resources.equator-network.org/reporting-guidelines/prisma/items/results-of-syntheses-statistical-results.html?utm_source=prisma&utm_medium=checklist&utm_campaign=1_1) | Present results of all statistical syntheses conducted. If meta-analysis was done, present for each the summary estimate and its precision (e.g. confidence/credible interval) and measures of statistical heterogeneity. If comparing groups, describe the direction of the effect. | Results – Review findings (p.16–18); Table 3 and Supplementary file S4 |
| [20c. Heterogeneity](https:/resources.equator-network.org/reporting-guidelines/prisma/items/results-of-syntheses-heterogeneity.html?utm_source=prisma&utm_medium=checklist&utm_campaign=1_1) | Present results of all investigations of possible causes of heterogeneity among study results. | Results – Review findings (p.16–18); Table 3 and Supplementary file S4 |
| [20d. Sensitivity analyses](https:/resources.equator-network.org/reporting-guidelines/prisma/items/results-of-syntheses-sensitivity-analyses.html?utm_source=prisma&utm_medium=checklist&utm_campaign=1_1) | Present results of all sensitivity analyses conducted to assess the robustness of the synthesized results. | Not performed |
| [21. Risk of reporting biases in syntheses](https:/resources.equator-network.org/reporting-guidelines/prisma/items/risk-of-reporting-biases-in-syntheses.html?utm_source=prisma&utm_medium=checklist&utm_campaign=1_1) | Present assessments of risk of bias due to missing results (arising from reporting biases) for each synthesis assessed. | Results – Review findings (p.16–18); Table 3 and Supplementary file S4 |
| [22. Certainty of evidence](https:/resources.equator-network.org/reporting-guidelines/prisma/items/certainty-of-evidence.html?utm_source=prisma&utm_medium=checklist&utm_campaign=1_1) | Present assessments of certainty (or confidence) in the body of evidence for each outcome assessed. | Discussion - Strengths and limitations (p.24-25) |
| **Discussion** |  |  |
| 23. Discussion |  |  |
| [23a. General interpretation of the results](https:/resources.equator-network.org/reporting-guidelines/prisma/items/discussion-general-interpretation.html?utm_source=prisma&utm_medium=checklist&utm_campaign=1_1) | Provide a general interpretation of the results in the context of other evidence. | Discussion (pp.19–27) |
| [23b. Limitations of included evidence](https:/resources.equator-network.org/reporting-guidelines/prisma/items/discussion-limitations-of-included-evidence.html?utm_source=prisma&utm_medium=checklist&utm_campaign=1_1) | Discuss any limitations of the evidence included in the review. | Discussion - Strengths and limitations (p.24-25) |
| [23c. Limitations of the review processes](https:/resources.equator-network.org/reporting-guidelines/prisma/items/discussion-limitations-of-review-process.html?utm_source=prisma&utm_medium=checklist&utm_campaign=1_1) | Discuss any limitations of the review processes used. | Discussion - Strengths and limitations (p.24-25) |
| [23d. Implications](https:/resources.equator-network.org/reporting-guidelines/prisma/items/discussion-implications.html?utm_source=prisma&utm_medium=checklist&utm_campaign=1_1) | Discuss implications of the results for practice, policy, and future research. | Discussion – Implications for practice, policy and research (p.26–27) |
| **Other Information** |  |  |
| 24. Registration and Protocol |  |  |
| [24a. Registration](https:/resources.equator-network.org/reporting-guidelines/prisma/items/registration-and-protocol-registration.html?utm_source=prisma&utm_medium=checklist&utm_campaign=1_1) | Provide registration information for the review, including register name and registration number, or state that the review was not registered. | Methods – Design and registration (p.7) |
| [24b. Protocol](https:/resources.equator-network.org/reporting-guidelines/prisma/items/registration-and-protocol-protocol.html?utm_source=prisma&utm_medium=checklist&utm_campaign=1_1) | Indicate where the review protocol can be accessed, or state that a protocol was not prepared. | Methods – Design and registration (p.7) |
| [24c. Amendments](https:/resources.equator-network.org/reporting-guidelines/prisma/items/registration-and-protocol-amendments.html?utm_source=prisma&utm_medium=checklist&utm_campaign=1_1) | Describe and explain any amendments to information provided at registration or in the protocol. | Methods – Deviations from protocol (p.8) |
| [25. Support](https:/resources.equator-network.org/reporting-guidelines/prisma/items/support.html?utm_source=prisma&utm_medium=checklist&utm_campaign=1_1) | Describe sources of financial or non-financial support for the review, and the role of the funders or sponsors in the review. | Funding statement (Title page) |
| [26. Competing Interests](https:/resources.equator-network.org/reporting-guidelines/prisma/items/competing-interests.html?utm_source=prisma&utm_medium=checklist&utm_campaign=1_1) | Declare any competing interests of review authors. | Declaration of competing interests |
| [27. Availability of data, code, and other materials](https:/resources.equator-network.org/reporting-guidelines/prisma/items/availability-of-materials.html?utm_source=prisma&utm_medium=checklist&utm_campaign=1_1) | Report which of the following are publicly available and where they can be found: template data collection forms; data extracted from included studies; data used for all analyses; analytic code; any other materials used in the review. | Data availability statement (and supplementary files) |

## 1 How to specify where content is

Tell the reader where they can find information. E.g.,

- Results; paragraph 2
- Methods, Participants; paragraphs 1 & 2.
- Table 3
- Supplement B, para. 4

If you have chosen not to describe an item, explain why. You can do this in the checklist, or as a note below it.

You can describe items in the article body, or in tables, figures, or supplementary materials, and should prioritize items you feel are most important to your intended audience. The order of items in your manuscript does not need to match the order of items in this checklist. You can decide how best to structure your work.

## 2 How to cite

Describe how you used PRISMA 2020 at the end of your Methods section, referencing the resources you used e.g.,

‘We used the PRISMA 2020 reporting guideline(1) to draft this manuscript, and the PRISMA 2020 reporting checklist(2) when editing, included in supplement A’

If you use a reporting checklist, remember to include it as a supplement when publishing so that readers can easily find information and see how you have interpreted the guidance.

1.	Page MJ, McKenzie JE, Bossuyt PM, Boutron I, Hoffmann TC, Mulrow CD, et al. The PRISMA 2020 statement: An updated guideline for reporting systematic reviews. PLOS Medicine [Internet]. 2021 Mar;18(3):e1003583. Available from: <https://journals.plos.org/plosmedicine/article?id=10.1371/journal.pmed.1003583>

2.	Page MJ, McKenzie JE, Bossuyt PM, Boutron I, Hoffmann TC, Mulrow CD, et al. The PRISMA 2020 reporting checklist. In: Harwood J, Albury C, Beyer J de, Schlüssel M, Collins G, editors. The EQUATOR network reporting guideline platform [Internet]. The UK EQUATOR Centre; 2025. Available from: [https:/resources.equator-network.org/reporting-guidelines/prisma/prisma-checklist.docx](https://https:/resources.equator-network.org/reporting-guidelines/prisma/prisma-checklist.docx)
